# A hierarchical sparse coding model predicts acoustic feature encoding in both auditory midbrain and cortex

**DOI:** 10.1371/journal.pcbi.1006766

**Published:** 2019-02-11

**Authors:** Qingtian Zhang, Xiaolin Hu, Bo Hong, Bo Zhang

**Affiliations:** 1 Department of Computer Science and Technology, Tsinghua University, Beijing, China; 2 Center for Brain-Inspired Computing Research (CBICR), Tsinghua University, Beijing, China; 3 School of Medicine, Tsinghua University, Beijing, China; University of California at Berkeley, UNITED STATES

## Abstract

The auditory pathway consists of multiple stages, from the cochlear nucleus to the auditory cortex. Neurons acting at different stages have different functions and exhibit different response properties. It is unclear whether these stages share a common encoding mechanism. We trained an unsupervised deep learning model consisting of alternating sparse coding and max pooling layers on cochleogram-filtered human speech. Evaluation of the response properties revealed that computing units in lower layers exhibited spectro-temporal receptive fields (STRFs) similar to those of inferior colliculus neurons measured in physiological experiments, including properties such as sound onset and termination, checkerboard pattern, and spectral motion. Units in upper layers tended to be tuned to phonetic features such as plosivity and nasality, resembling the results of field recording in human auditory cortex. Variation of the sparseness level of the units in each higher layer revealed a positive correlation between the sparseness level and the strength of phonetic feature encoding. The activities of the units in the top layer, but not other layers, correlated with the dynamics of the first two formants (F1, F2) of all phonemes, indicating the encoding of phoneme dynamics in these units. These results suggest that the principles of sparse coding and max pooling may be universal in the human auditory pathway.

## Author summary

When speech enters the ear, it is subjected to a series of processing stages prior to arriving at the auditory cortex. Neurons acting at different processing stages have different response properties. For example, at the auditory midbrain, a neuron may specifically detect the onsets of a frequency component in the speech, whereas in the auditory cortex, a neuron may specifically detect phonetic features. The encoding mechanisms underlying these neuronal functions remain unclear. To address this issue, we designed a hierarchical sparse coding model, inspired by the sparse activity of neurons in the sensory system, to learn features in speech signals. We found that the computing units in different layers exhibited hierarchical extraction of speech sound features, similar to those of neurons in the auditory midbrain and auditory cortex, although the computational principles in these layers were the same. The results suggest that sparse coding and max pooling represent universal computational principles throughout the auditory pathway.

## Introduction

Hearing is supported by a series of interconnected brain areas, collectively called the central auditory system or auditory pathway [1]. This pathway is thought to function as a series of hierarchical processing stages that encode features ranging from simple acoustic features and elementary time–frequency representations in the cochlea and inferior colliculus to complex phonetic features, phonemes, syllables, words, and grammatical features in the auditory cortex [2–8]. However, it remains unclear how neurons encode these distinctive features, especially at higher stages.

The encoding mechanisms of neurons in the auditory pathway have been addressed in many studies, and various encoding mechanisms have been proposed. These include spatial coding in the cochlea [9–11], spatial coding and temporal coding in the inferior colliculus [12–16], and spatial coding and periodicity coding in the auditory cortex [17–19]. However, these studies only describe the experimental data, and do not explain why the experiments yield specific outcomes. A notable exception is the sparse coding model, which assumes that neurons encode external stimuli using sparse codes. The model was originally proposed to explain the properties of simple cells in the primary visual cortex [20, 21], but has been extended to explain the emergence of the response properties of auditory nerve fibers [22] and inferior colliculus neurons [23]. Another exception is a deep learning model that has been used to explain the emergence of response properties of neurons to speech in the auditory cortex [24]. Because the model was trained to recognize 40 English phonemes in a supervised fashion, high discrimination ability is critical for its success. These two models effectively explained certain neural response properties at different stages along the auditory pathway; however, their underlying computational principles are different. A fundamental question is whether the auditory system uses the same or different principles at different stages. We explored the former possibility in this study.

Our initial assumption was that sparse coding plays an important role in shaping neural response properties along the auditory pathway. Support for this is provided by the ubiquitous sparse firing of neurons in the auditory pathway [25–27], and sparse coding computational models have effectively interpreted experimental data recorded at certain stages of the auditory system [22, 23]. We extended this model to multiple layers by introducing spatial pooling after each layer, resulting in an unsupervised deep learning model, which we named sparse HMAX (SHMAX) [28]. We studied the response properties of the computing units, called *artificial neurons*, in the model. After training the model on the cochleogram of speech, the spectro-temporal receptive fields (STRFs) of artificial neurons in lower layers in the model exhibited patterns similar to those of neurons in the inferior colliculus, whereas the responses of artificial neurons in the upper layers resembled the results of field recordings in human auditory cortex. The agreement between the model and neural data suggest that, although the features encoded at different levels of the auditory pathway differ, the encoding mechanisms are similar.

## Results

**Fig 1:**
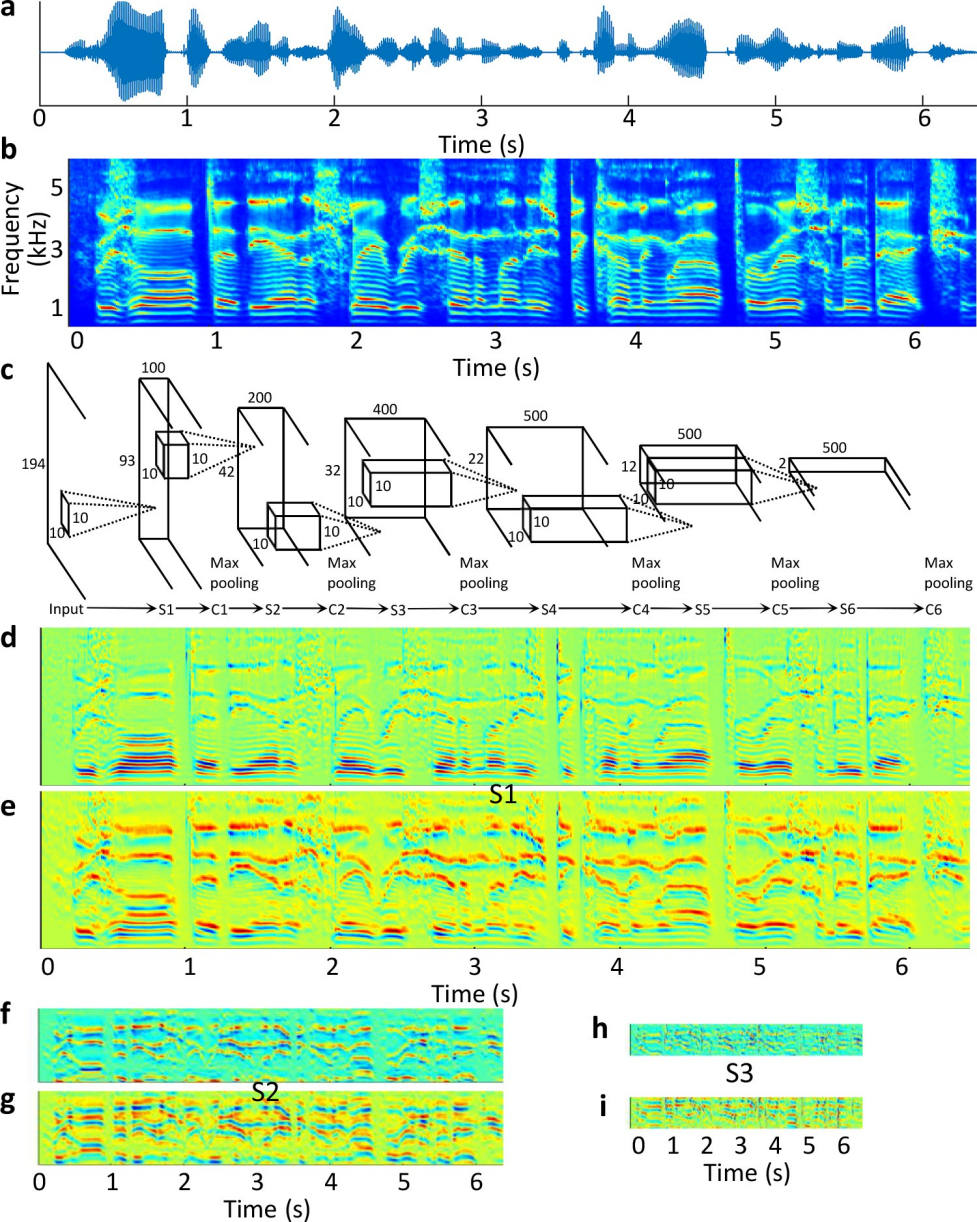
Stimuli and experimental protocol. (a) Example stimulus. (b) Cochleogram of the example stimulus. (c) Structure of SHMAX, which consists of alternate sparse coding layers (S layers) and max pooling layers (C layers). To avoid clutter, only S layers are displayed. The height of the feature maps in each S layer is indicated on the left, and the number of feature maps in each S layer is indicated at the top. The width of the feature maps (the temporal dimension) is not indicated because it varies according to the length of the input sentence. (d, e) Two example feature maps (activations of two features in response to the example stimulus) in layer S1. (f, g) Two example feature maps in layer S2. (h, i) Two example feature maps in layer S3.

A hierarchical sparse coding model, SHMAX [28], was trained on the cochleogram of English speech, which consisted of six S layers and six C layers in alternation (i.e., S1-C1-S2-C2…; Fig 1C). The S layers perform sparse coding and the C layers perform max pooling to integrate feature specificity and invariance, respectively, both of which are important for recognition. Each layer has multiple feature maps (Fig 1D–1I), and each feature map consists of the responses of artificial neurons with the same receptive field shape but different displacements in the input space. In the model, the number of feature maps increases from 100 in layers S1 and C1 to 500 in layers S6 and C6. This setting does not reflect biological facts, but is intended simply to balance the computing resource consumption in different layers because the feature maps are larger in lower layers and smaller in higher layers. The results obtained with other settings would be similar to those presented here if the number of feature maps in each layer was large enough.

We hypothesized that lower layers correspond to lower-level stages in the auditory pathway such as the inferior colliculus, whereas higher layers correspond to higher-level stages such as the auditory cortex. We compared the response properties of the artificial neurons in different layers to those of real neurons in the inferior colliculus and auditory cortex.

### Response properties of the lower-level units resembled those of the auditory midbrain neurons

We first confirmed that the computing units in lower layers of the model could capture the firing properties of the inferior colliculus neurons in the auditory midbrain. This capability has been proven in a single-layer sparse coding model [23]; however, the time window of the bases in that model was too large (216 ms), and it was unclear whether a much smaller time window such as 20 ms, which is more compatible with physiological data [29, 30], would yield similar results. In addition, an overly large time window in the first layer causes difficulty in constructing deep models so that the time windows of higher-layer units agree with those of cortical neurons. Our model started with a time window of 10 ms in layer S1 and ended with a time window of 194 ms in layer C6. In the following, we will show that, with these settings, several lower layers of the model, rather than only the lowest layer, can generate results qualitatively similar to those obtained in the previous study [23].

**Fig 2:**
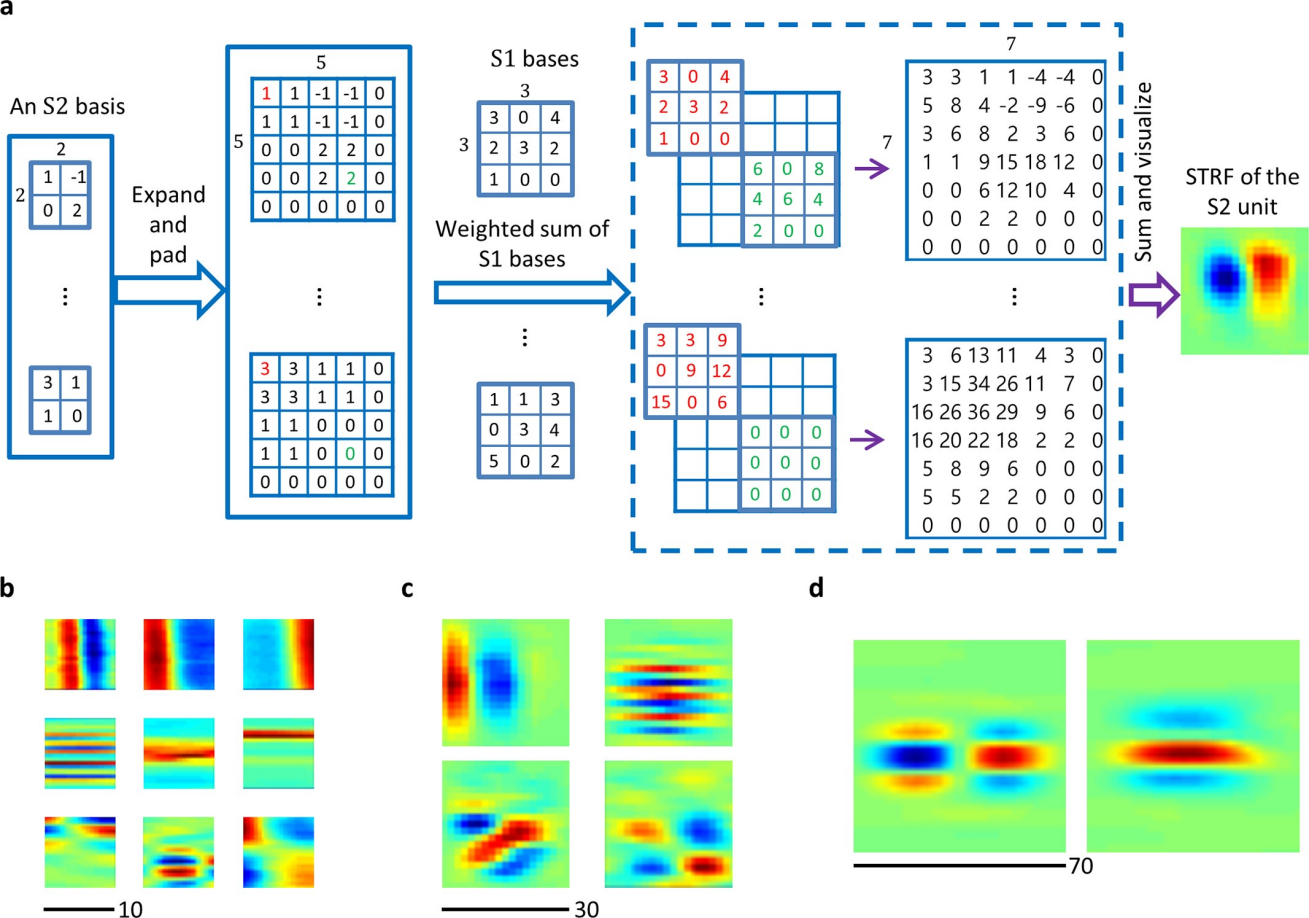
Calculation of STRFs and example STRFs. (a) Illustration of the visualization of an S2 unit whose basis has size 2 × 2 × *u*^1^, where *u*^1^ denotes the total number of S1 bases. The size of each S1 basis is 3 × 3. Suppose that there is a down-sampling operation with ratio 2 between layer S1 and layer S2, which could be a convolution with stride 2 in layer S2 (the case in this study) or a max pooling with ratio 2 and stride 2. In that case, we first need to expand each slice of the S2 unit, a 2 × 2 matrix, to a 4 × 4 matrix. Because there is a max pooling layer with pooling ratio 2 and stride 1 between layers S1 and S2, the first two dimensions of the S2 feature maps are 1 smaller than those of the S1 feature maps. To account for this effect, we pad zeros around the 4 × 4 matrices to obtain 5 × 5 matrices. Each 5×5 slice can be viewed as learned on the feature map, which is obtained by convolving an S1 basis on its previous layer, the input image. Then, the effect of this 5 × 5 slice in layer S1 is roughly equivalent to that of a 7 × 7 matrix (shown on the right in the dashed box) formed by summing the same S1 basis centered at 25 locations and weighted by the corresponding elements in the slice. For illustration, on the left in the dashed box, the sum of the S1 basis weighted by two elements (red and green) in each slice is shown. The STRF of the example S2 unit is the sum of all *u*^1^ 7 × 7 matrices. (b) Example STRFs in layer S1. (c) Example STRFs in layer S2. (d) Example STRFs in layer S3.

The response properties of an inferior colliculus neuron are usually delineated by STRFs, which are obtained by averaging the spectro-temporal structure of acoustic stimuli before a spike is fired [31]. The STRFs of the S1 units can be approximated by the bases used to reconstruct the stimuli [23]. To visualize the STRFs of higher-level units, we linearly combined the bases of units in previous layers (see Materials and Methods; Fig 2A; S1 Fig). Several example STRFs of units in the first three S layers are shown in Fig 2B–2D, and the full results are shown in S2 Fig. This visualization method is simple, fast, and applicable to lower-layer units. In fact, the results are similar to the STRFs obtained by the normalized reverse-correlation method [32, 33] (see Materials and Methods; S3 Fig). However, in deeper layers, the STRFs become larger and more complex, and exhibit stronger nonlinearity, and are therefore difficult to capture with a linear method.

**Fig 3:**
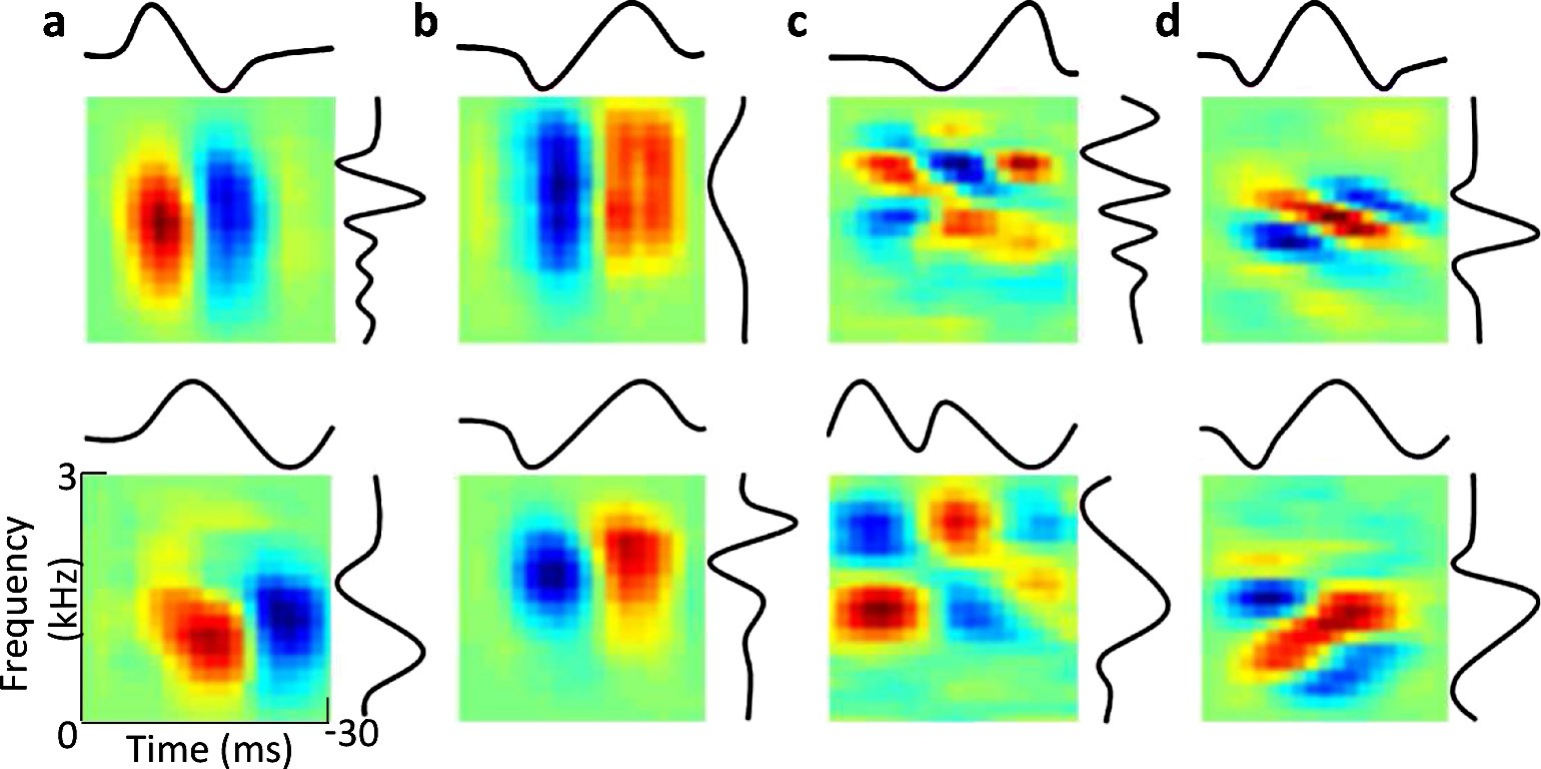
Visualization of the representative bases in layer S2 along with typical STRFs of the inferior colliculus neurons in animals. (a–d) STRFs of several typical layer S2 units. Curves denote the spectral and temporal profiles obtained by SVD. (a) Two ON-type units. (b) Two OFF-type units. (c) Two localized checkerboard units. (d) Two spectral motion units. Similar STRFs of typical inferior colliculus neurons have been observed in physiological experiments. One can compare (a) with Fig 3E in [34], (b) with Fig 6A in [23], (c) with Fig 7A in [23] and (d) with Fig 6C in [29].

By visual inspection, all of the first three S layers exhibited a certain degree of agreement with physiological data collected from the inferior colliculus, with layer S2 providing the best match in terms of STRF size and pattern (a quantitative comparison will be provided later). Representative bases of layer S2 units are visualized in Fig 3. Most layer S2 bases had both excitatory regions and inhibitory regions. Specifically, some units favored excitation first, followed by inhibition at the same frequency (Fig 3A). This pattern has been observed in STRFs of inferior colliculus neurons in cats [34]. By contrast, some layer S2 units favored inhibition first, followed by excitation at the same frequency (Fig 3B). This pattern has been observed in STRFs of inferior colliculus neurons in gerbils [23, 35]. Some layer S2 bases had localized checkerboard patterns in their STRFs (Fig 3C), also as in inferior colliculus neurons in gerbils [23, 35]. Some layer S2 units were selective to spectral motion (Fig 3D), as in certain inferior colliculus neurons in Mexican free-tailed bat that are tuned to motion cues present in conspecific vocalizations [29]. In those studies, the favored frequencies of layer S2 bases differed from those of inferior colliculus neurons because the experiments were carried out on gerbils and cats, whereas our deep learning model was trained on human speech data with a lower range of frequency content.

**Fig 4:**
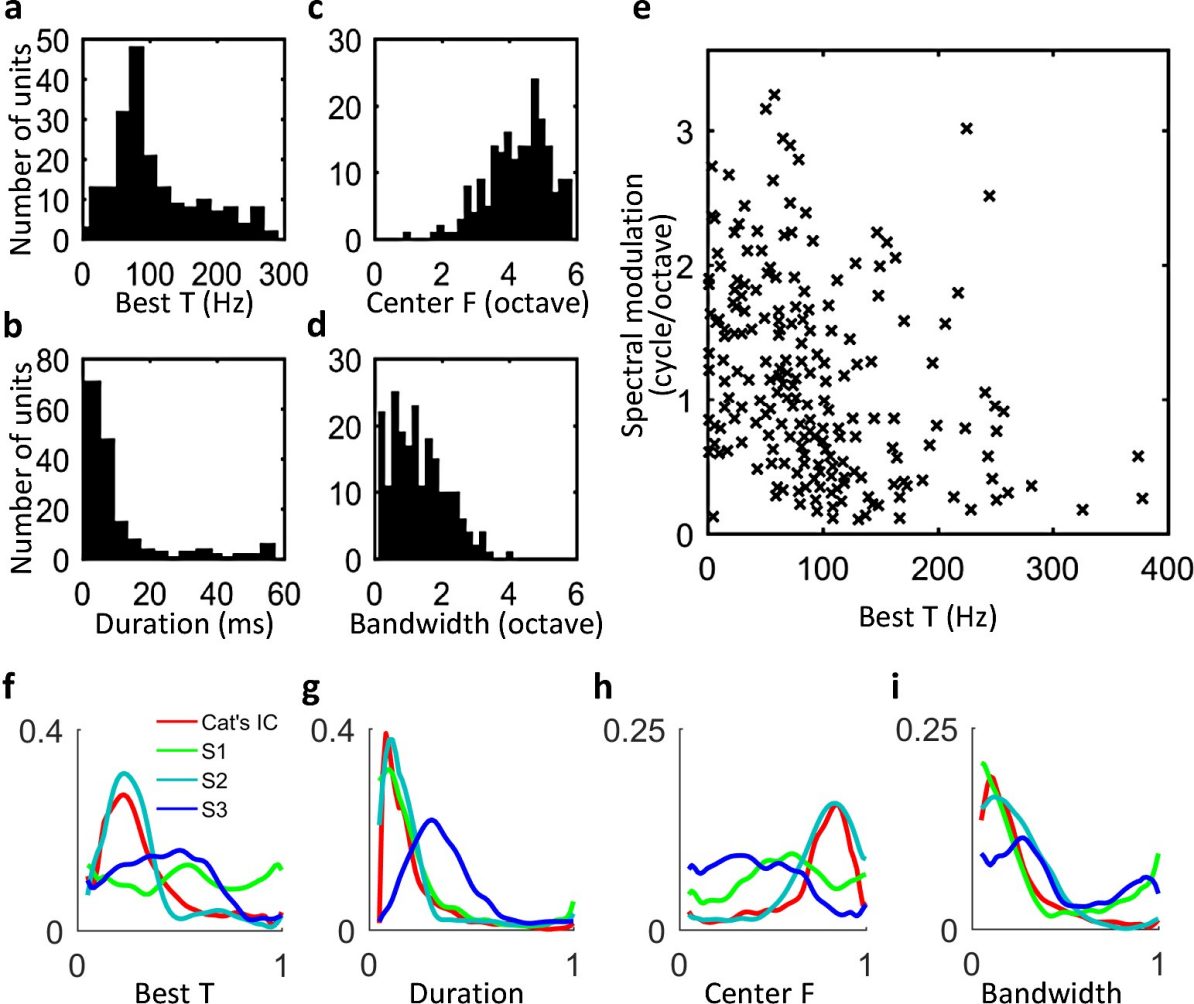
Distributions of STRF parameters of layer S2 units. (a) Best temporal modulation frequency. (b) Response duration. (c) Center frequencies. (d) Spectral bandwidth. These four parameters respectively correspond to the peak and bandwidth with 90% power of the temporal and spectral profiles shown in Fig 3. (e) Tradeoff between temporal modulation (Best T) and spectral modulation. (f–i) Probability distribution of STRF parameters normalized from the corresponding histograms. For comparison, the normalized probability distributions in layers S1 and S3 and the reference distributions of inferior colliculus neurons in cats [30] are also plotted. The horizontal axis in each panel is normalized to [0, 1] by dividing all values by the maximum value.

We calculated the distributions of four parameters that characterized a STRF over all layer S1, S2, and S3 units, separately: best temporal modulation frequency (Best T), response duration (Duration), center frequency (Center F), and spectral bandwidth (Bandwidth). Fig 4A–4D shows the results of layer S2 units. The shapes of the distributions of layer S2 units were most similar to those of inferior colliculus neurons in cats [30] (Fig 4F–4I). The scatterplot of Best T and spectral modulation (Fig 4E) indicated a tradeoff between the temporal modulation and spectral modulation among layer S2 units; i.e., units with both high temporal modulation (Best T) and spectral modulation were scarce. This result agrees with observations made in inferior colliculus neurons of cats [34].

### Response properties of higher-layer units resembled those of field recordings in human auditory cortex

**Fig 5:**
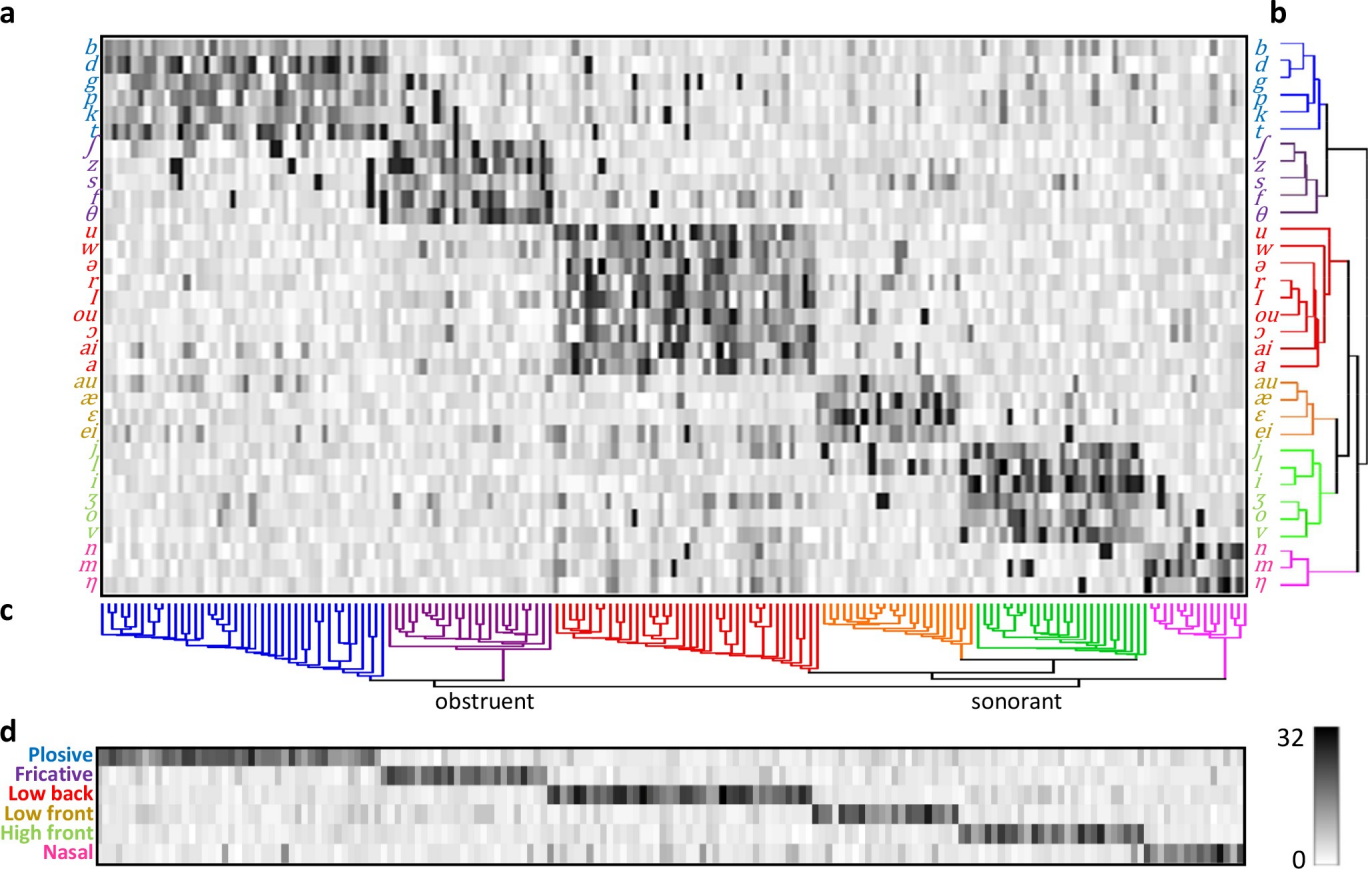
PSI vectors of 173 active units in layer C6. (a) PSI vectors of phonemes. Each column corresponds to a unit. (b) Hierarchical clustering across phonemes. (c) Hierarchical clustering across units. (d) PSI vectors of six phonetic features.

We hypothesized that the higher layers in the network would correspond to the auditory cortex. A previous study using cortical surface recordings in humans reported selectivity for distinct English phonetic features at single electrodes [6]. Hence, we investigated whether similar results could be obtained in higher layers of our network. Following that study [6], we separately calculated the phoneme selectivity index (PSI) vectors of the units in layers S1 to C6 (see Materials and Methods). Each element in the PSI vector of a unit indicates the selectivity of the unit to a phoneme; the larger the element, the more selective to the corresponding phoneme. Units in lower layers, such as S1 to S4, did not exhibit distinctive phoneme selectivity (S4 Fig), whereas those in higher layers did (S5 Fig; Fig 5). As an example, Fig 5A shows the PSI vectors of 173 active units in layer C6 whose responses to randomly selected time frames were statistically larger than their response to silence (p<0.001). Each of these units exhibits strong selectivity for a subset of phonemes, and the unit–phoneme map exhibits a strong clustering effect. We used hierarchical agglomerative clustering analyses [36] with Euclidean distance to determine selectivity patterns across phonemes (Fig 5B) and active units (Fig 5C). Phonemes were clustered into six groups according to the place and manner of articulation: plosive, fricative, low back, low front, high front, and nasal. The active units were also clustered into six groups, each selective for one of the six types of phonemes. The PSI vectors of active units sharing a particular phonetic feature were averaged to quantify the feature selectivity of these units. Six groups of units exhibited distinctive roles in characterizing these features (Fig 5D).

Similar clustering results were obtained in layers S5, C5, and S6 (S5 Fig). However, the dark area in the unit–phoneme plane becomes increasingly prominent from layer S5 to layer C6 (S5 Fig; Fig 5), suggesting increasing selectivity of these layers for phonetic features. As in previous studies [24, 37], we defined an index (F-ratio) that measures the overall selectivity of each hidden layer to phonetic features (Materials and Methods). We found that the deeper the layer, the higher the F-ratio (Table 1). Specifically, the active units in layer C6 exhibited the highest overall selectivity.

**Table 1:** F-ratios of the last eight layers.

Layer	S3	C3	S4	C4	S5	C5	S6	C6
F-ratio	3.28	3.31	6.16	8.82	13.80	18.74	61.35	73.41

**Fig 6:**
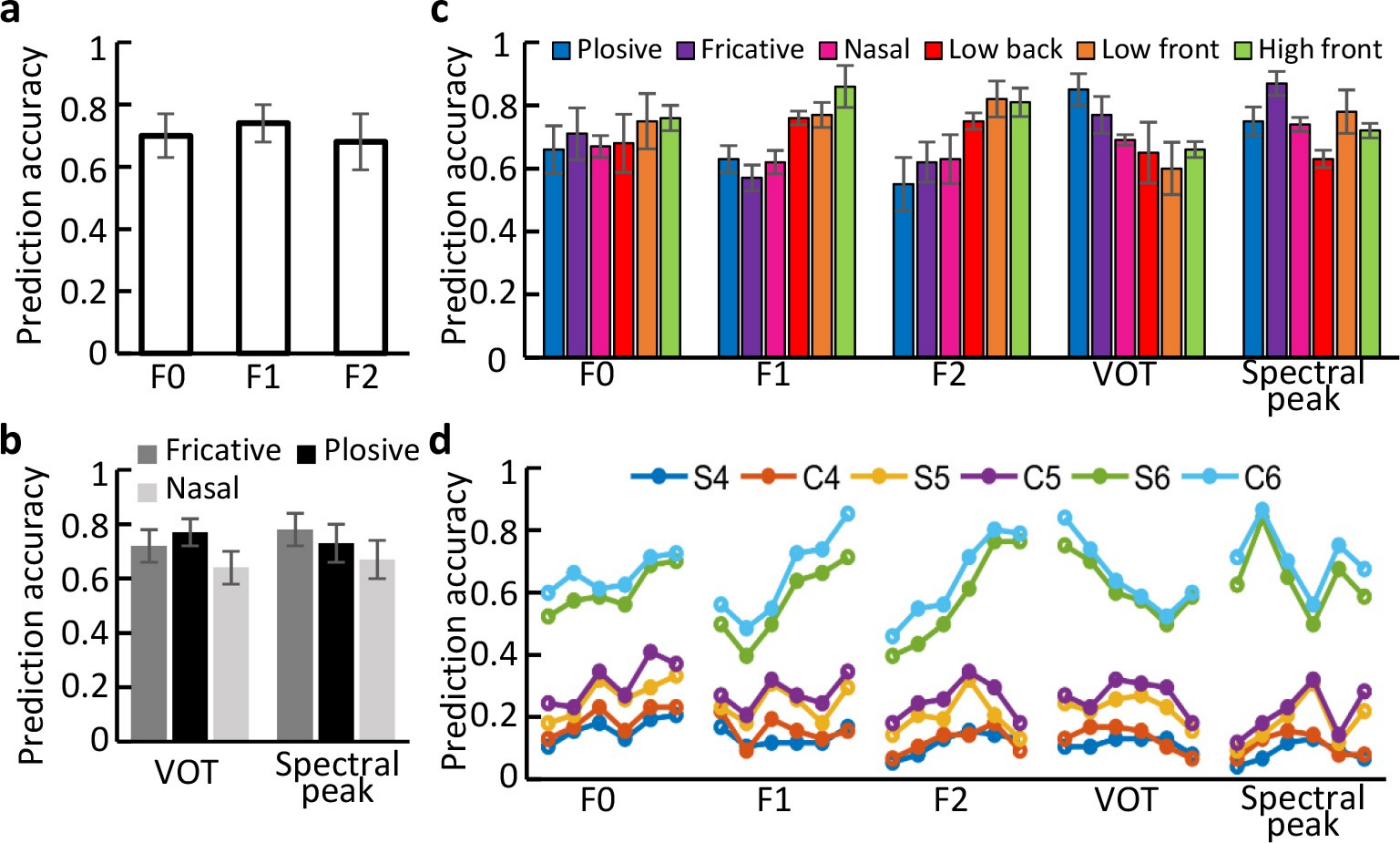
Encoding of the acoustic parameters F0, F1, F2, VOT, and spectral peak in higher layers. The mean and standard deviation of decoding accuracies in 20-fold training and testing experiments are shown. (a) Decoding accuracies of F0, F1, and F2 based on the response amplitudes of all active layer C6 units. These accuracies are significantly higher than that of a random decoder (p<10^−5^). (b) Decoding accuracies of VOT and spectral peak based on the response amplitudes of all active layer C6 units. These accuracies are significantly higher than that of a random decoder (p<10^−5^). (c) Decoding accuracies of acoustic parameters based on the response amplitudes of active layer C6 units in six different groups. These accuracies are significantly higher than that of a random decoder (p<10^−5^). (d) Average decoding accuracies of the acoustic parameters in layers S4, C4, S5, C5, S6, and C6. The six groups of units are presented in the same order as in (c) (from left to right: plosive, fricative, nasal, low back, low front, and high front). In all panels, error bars indicate standard deviation over 20 accuracies. To avoid clutter, error bars in (d) are not shown.

Phonetic feature categories are discrete acoustic parameters. We next investigated the encoding of continuous acoustic features that specify phonemes, including the fundamental frequency (F0), formant frequencies (F1, F2), voice-onset time (VOT), and spectral peak. We used linear regression to decode these features from the response amplitudes of model units (Materials and Methods). Because F0, F1, and F2 vary significantly across vowels, whereas VOT and spectral peak vary significantly across consonants, we separately decoded F0, F1, and F2 of vowels (Fig 6A) and the VOT and spectral peak of consonants (Fig 6B) from the responses of all active units in layer C6. A 20-fold validation scheme was used to predict each parameter. The prediction accuracies on the test sets (1-fold) were defined based on the regression errors on the corresponding training sets (19-fold). The prediction accuracies for each parameter were significantly higher than those of a random decoder (p<10^−5^; Materials and Methods). These observations suggest that the variability of these acoustic features is well represented in the responses of the units. Similar prediction accuracies were obtained based on the neural population responses in human superior temporal gyrus [6].

The same linear regression method was applied to decode the acoustic parameters F0, F1, F2, VOT, and spectral peak from the cochleogram. We cut a length of 170 ms cochleogram for each phoneme instance and used it as the feature of this instance. All decoding accuracies were about 10%, much lower than those obtained from the responses of layer C6 units (Fig 6A and 6B).

In higher layers of the model, the units were always clustered into six phoneme groups according to PSI (Fig 5; S5 Fig). Therefore, we could calculate the decoding accuracies for each group of units and compare the results in different layers. In layer C6, we obtained significantly higher accuracies than did a random decoder (Fig 6C) (p<10^−5^). Similar results were obtained in layer S6 (Fig 6D). However, the decoding accuracies were very low in layers S5 and C5, and even lower in the earlier layers S4 and C4. These poor accuracies were partly due to the small STRFs of units in these layers, which contain less information about the acoustic features of a phoneme. Fig 6D shows a small improvement from layer S(*l*) to layer C(*l*), but a large improvement from layer C(*l*) to layer S(*l*+1). This is because STRF sizes were similar between units in layer S(*l*) and layer C(*l*), but very different between units in layer C(*l*) and layer S(*l*+1) (Table 2).

**Table 2:** STRF sizes of the units in different layers.

Layer	S1	C1	S2	C2	S3	C3	S4	C4	S5	C5	S6	C6
STRF Size	10	12	30	34	70	74	110	114	150	154	190	194

**Fig 7:**
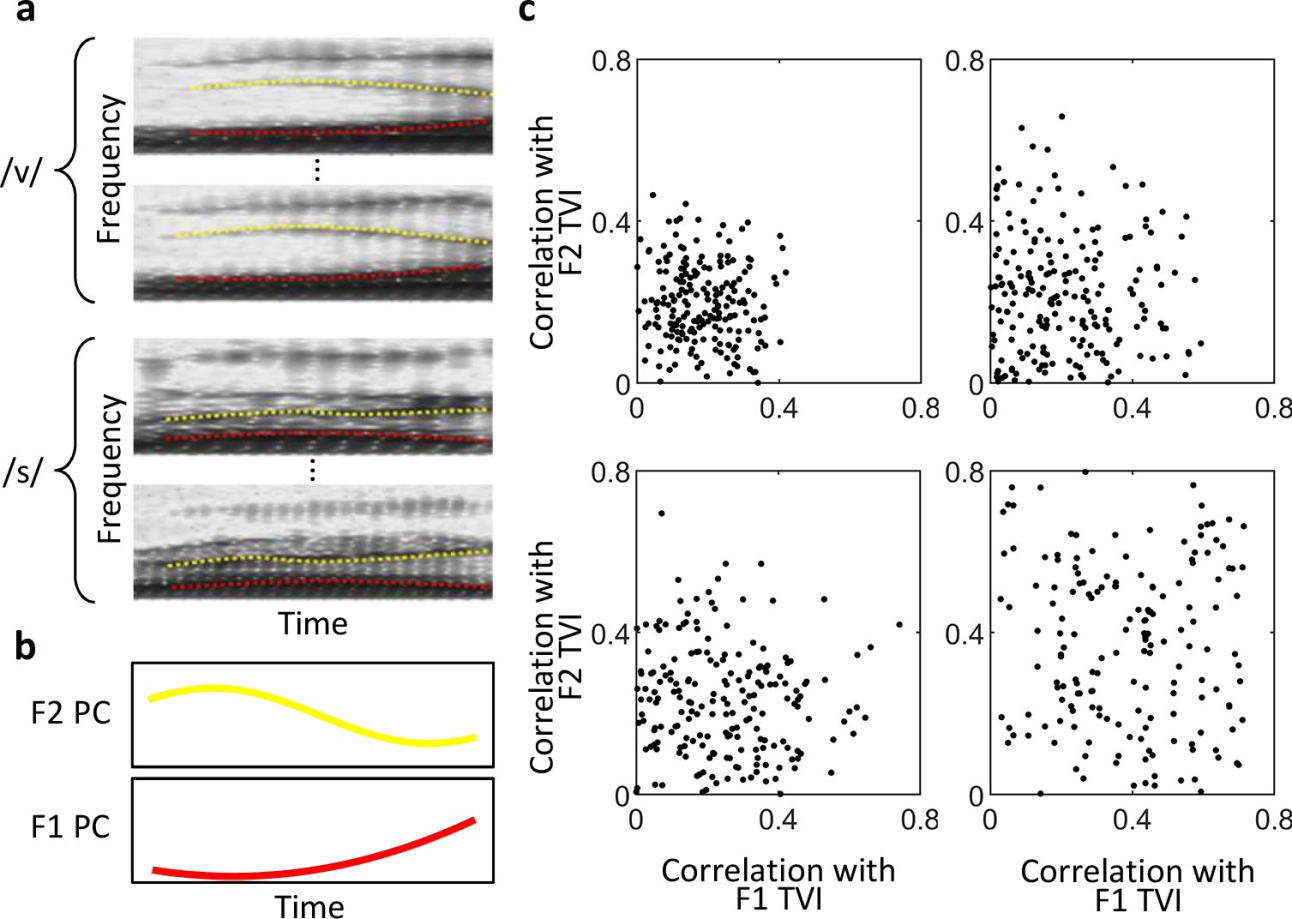
Encoding of dynamic properties of phonemes in higher layers. (a) Spectrograms of two phonemes. Two instances of each phoneme are shown. The first two formant (F1 and F2) contours of these instances are denoted by red and yellow curves, respectively. The formant contours of a phoneme was defined as the averaged contours of different instances of the phoneme. (b) Principal components (PCs) of the F1 and F2 contours calculated over 33 phonemes. F1 or F2 TVI of a phoneme is defined as the projection of the phoneme’s F1 or F2 contour onto the F1 or F2 PC. (c) Encoding of the dynamic properties of phonemes in different layers. Each dot indicates the correlations between the responses of a unit to the phonemes and their F1 (horizontal axis) and F2 (vertical axis) TVIs. In each layer, 200 units were randomly selected.

Thus far, we have described the encoding of static acoustic features of phonemes in higher layers of the model. However, phonemes are not static. In fact, the first two formants (F1 and F2) of the phonemes, especially the consonants, exhibited large variation over time (Fig 7). We investigated the encoding of the dynamic formants using the responses of the units. We defined the temporal variation index (TVI) of individual phonemes as the projections of their F1 or F2 contours (time course of formant frequencies averaged over all instances of a phoneme) onto their respective principal components over all phonemes (Materials and Methods). Therefore, TVI measures the matching degree of an individual formant contour to the principal component of all formant contours. For each unit, we calculated the correlations between its responses to phonemes and the TVIs of the phonemes. A high correlation indicated that the unit was sensitive to the TVI of the phonemes—it “liked” phonemes whose F1 or F2 contour were congruent to the principal component but “disliked” phonemes whose F1 or F2 contour were incongruent to the principal component. Some units in layer C6 were sensitive to either F1 or F2 TVI, whereas others were sensitive to both F1 and F2 TVI (Fig 7C). However, such units were scarce in layers S5, C5, and S6; in fact, for most of the units in these layers, the correlations between their responses and both F1 and F2 TVI were smaller than 0.5.

### Influence of sparseness to response properties of higher-layer units

The hallmark of the sparse coding model [20, 38] is the sparse activity of the hidden units, which has been proven to be essential in reproducing the tuning properties of auditory nerve fibers [22] and neurons in the inferior colliculus [23]. However, it remains unclear whether sparse activity in higher layers of SHMAX also plays a significant role in producing the phoneme encodings that we observed. Hence, we investigated how the sparseness level influenced the results in layer S5 to layer C6.

**Fig 8:**
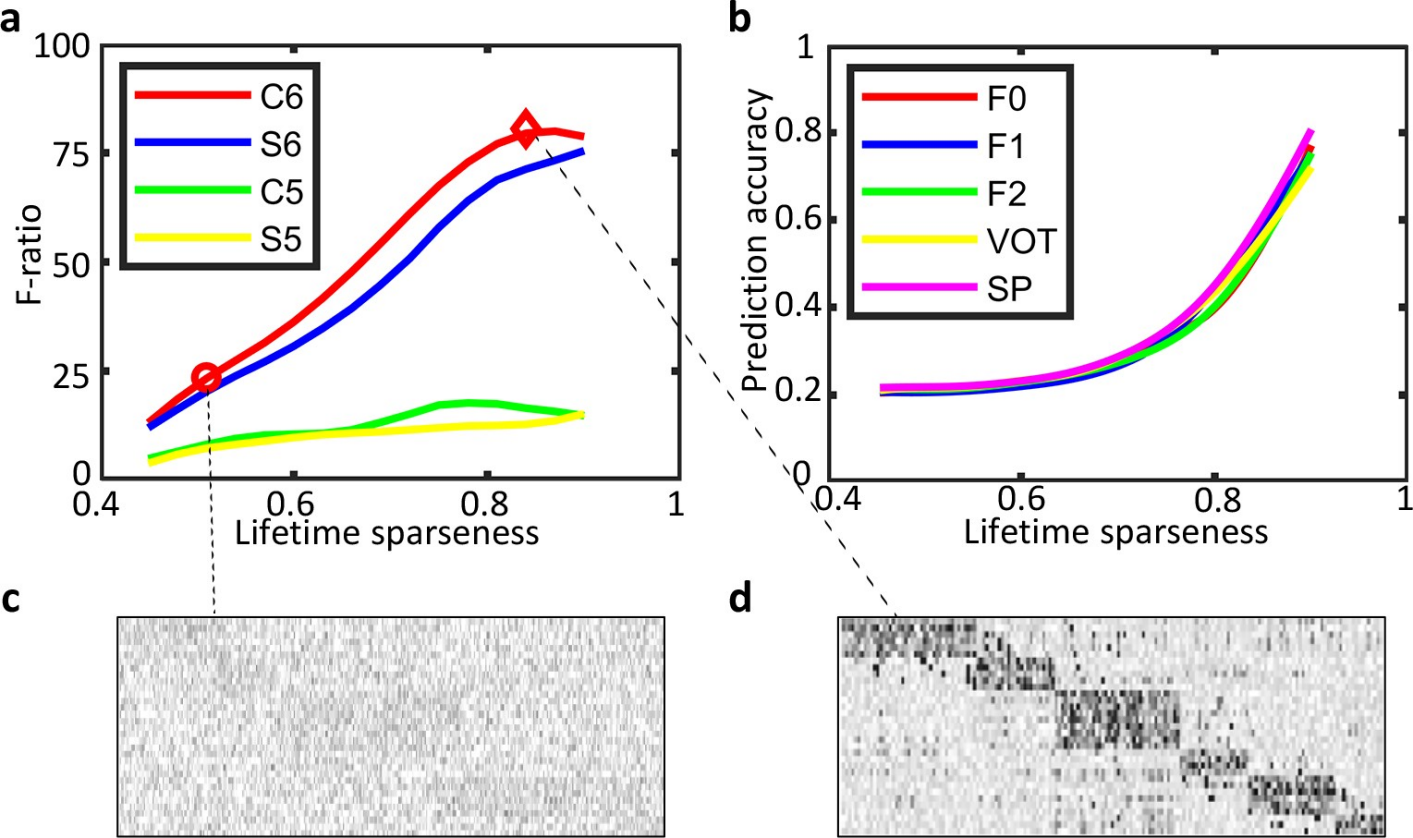
Influence of the response sparseness of the units in the model. To obtain the curve for one layer, a total of 16 values were chosen non-uniformly between 0.001 and 100 for λ in that layer, while keeping λ = 1 in lower layers. (a) Relationship between F-ratio and lifetime sparseness in layers S5, C5, S6, and C6. (b) Relationship between the decoding accuracy of different acoustic parameters and lifetime sparseness in layer C6. SP, spectral peak. (c) PSI vectors of phonemes in C6 with λ = 0.01. The order of rows is the same as in Fig 5A. (d) PSI vectors of phonemes in C6 with λ = 1 (exactly the same as Fig 5A).

First, we adjusted the parameter *λ* in the sparse coding model, which controls the sparseness of the responses of the units of a particular S layer, while keeping *λ* in other layers at the default value of 1. We found that sparseness and F-ratio were positively correlated in each layer (Fig 8A), i.e., the sparser the activity, the more selective the units. However, increasing the sparseness level in lower layers such as S5 and C5 did not lead to phoneme tuning as strong as that in C6. This suggests that sparseness was not the only factor that led to the strong phoneme-encoding property in the top layer, and that the hierarchical structure also played a significant role. Although we used the lifetime sparseness measure [39] (Materials and Methods) here, the conclusion did not change when the population sparseness measure [40] was applied. Second, we modulated the neural encoding of acoustic parameters with different sparseness levels. The results revealed that sparseness also played a key role in producing the neural population coding of acoustic parameters (Fig 8B).

### Influence of pooling on response properties

**Fig 9:**
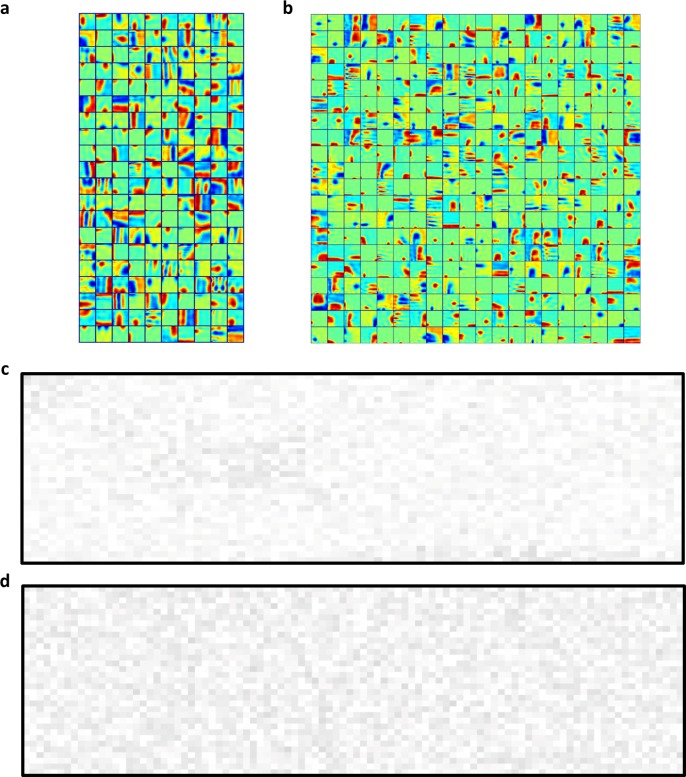
Influence of the pooling method used in the model. (a, b) STRFs of all units in layers S2 and S3 without pooling. (c) PSI vectors of 77 active units in layer S6 without pooling. (d) PSI vectors of 96 active units in layer C6 with average pooling.

The other critical element in the model is max pooling. Without this element, the model would be almost linear because the only nonlinear operation is the down-sampling between layer S1 and layer S2, which is implemented by a convolution with stride 2; one would not expect such a model to produce striking results. To examine the influence of pooling, first, we removed the max pooling layers and trained the model as before. Under these conditions, the STRFs of layer S2 and layer S3 units exhibited much simpler patterns (Fig 9A and 9B), and the complex patterns obtained with max pooling (Fig 3) were scarce. More importantly, the higher layers did not exhibit similar results to those of field recordings in human auditory cortex (e.g., compare Fig 9C with S5C Fig). Second, we replaced all max pooling in the model with average pooling, another widely used operation in the deep learning field. The only difference is that, in average pooling, we take the average of values, instead of the maximum value, in a region. After training the model, we obtained poor results (e.g., compare Fig 9D and Fig 5A). These results suggest that max pooling played an important role in modeling the function of the auditory pathway.

### Influences of the number of layers and STRF size

**Fig 10:**
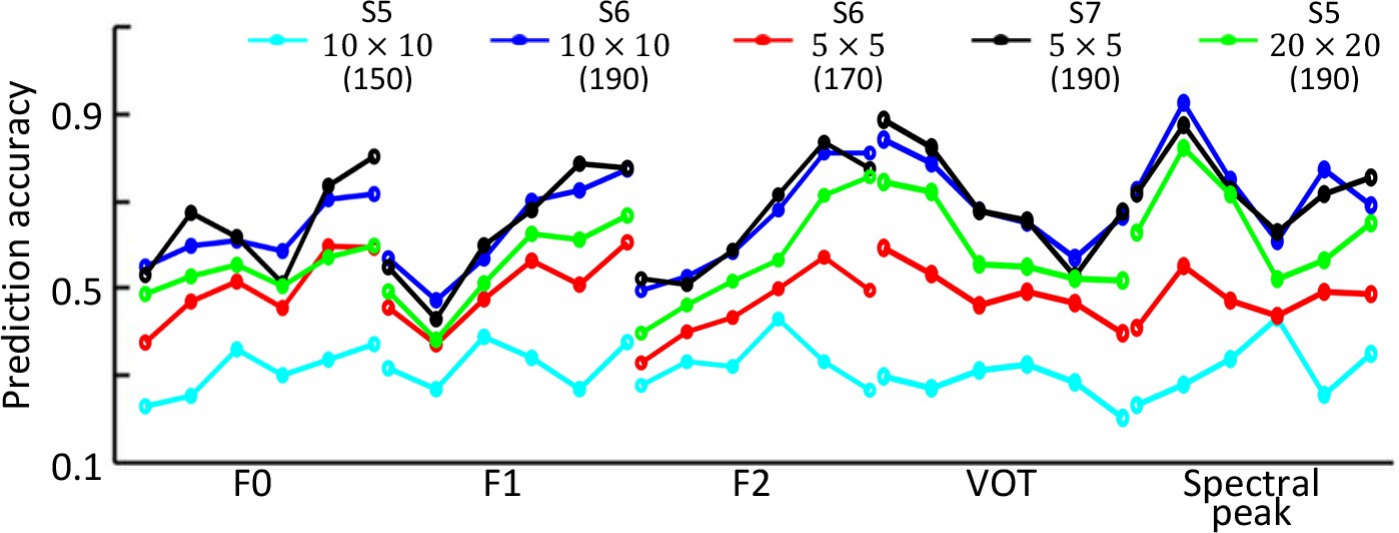
Average decoding accuracies of the acoustic parameters in layers S5, S6, and S7 with different kernel sizes. Note that S5 with kernel size 10×10 and S6 with kernel size 10×10 are layers in the original network. Layer S5 with kernel size 20×20 was obtained by fixing layers S1 to C4 of the original network; layer S6 with size 5×5 and layer S7 with kernel size 5×5 were obtained by fixing layers S1 to C5 of the original network. The STRF sizes in these layers are indicated in parentheses.

In addition to sparse response and max pooling, two other factors are also important for the emergence of phoneme selectivity: the number of layers and STRF size of the units. This is because enough nonlinearity must be accumulated along the hierarchy through repeated max pooling and down-sampling, and the STRF must be large enough to cover the length of the phonemes. To better understand the roles of these two factors, we explored models with different architectures. First, we tried a different layer S5 with larger kernel size (20×20), whose STRF size was the same as in the original layer S6. The selectivity of the new layer S5 was weaker than that of the original layer S6 (S6A Fig; Fig 10). Because the nonlinearity of the model grows with ascending layers, this result indicates that the emergence of phoneme selectivity needs a certain degree of nonlinearity. Second, we replaced the original layer S6 (kernel size 10×10) with two new layers, S6 and S7, with kernel size 5×5. In comparison with the original layer S6, the phoneme selectivity of the new layer S6 was weaker, and that of the new layer S7 was similar (S6B and S6C Fig; Fig 10). The results indicate that STRF size is also important for the emergence of phoneme selectivity. Notice that there is an abrupt increase in the decoding accuracy of acoustic parameters from original layer S5 to layer S6, as observed in Fig 6D. This is mainly because the STRFs of layer S5 units were not large enough to capture the selectivity information, while those of layer S6 units happened to be large enough. Fig 10 shows that, if the STRF size in original layer S5 is increased (green), or the STRF size in original layer S6 is decreased (red), the increase in decoding accuracy from the original layer S5 to layer S6 would not be as large as observed in Fig 6D.

## Discussion

In this study, we demonstrated the capability of a computational model to predict the neural response properties along the auditory pathway. With alternating sparse coding and max pooling operations, the model learned important features of speech, from simple spectro-temporal patterns to complex phonetic features, which are encoded at different stages of the auditory pathway. Specifically, the response properties of lower-layer units were similar to those of inferior colliculus neurons in the auditory midbrain, whereas the response properties of higher layers were similar to field recordings in the auditory cortex. It is worth emphasizing that the agreement between the model output and neural physiological data is not a result of neural data fitting, but rather of unsupervised learning of the deep network. Because the auditory system deals with dynamic sound stimulus and has more processing stages before the auditory cortex, its coding strategy was thought to be distinct from that of the visual system [41]. Our model demonstrated for the first time that the sparse and hierarchical coding strategies widely observed in the visual system can also be generalized to the auditory system.

### Sparse coding and max pooling in auditory computation

Merely reconstructing the stimuli without sparseness regularization would not lead to biologically reasonable results. Indeed, we observed that, upon replacing the L1 regularization term in Eq (1), which encourages sparse activities in the units, with the L2 regularization term $\lambda \sum_{k}{\left\|r^{k}\right\|}_{2}^{2}$, the STRFs of lower-layer units had fewer semantic features, and the phonetic feature selectivity was poor even at layer C6. This result agrees with a recent study [37] in which two unsupervised deep neural networks trained on natural speech without this regularization exhibited no clear selectivity for phonetic features.

A previous study [23] using a single-layer sparse coding model reported qualitatively similar results to those obtained from the lower layers of SHMAX. The work described here extends that study in two ways: first, by setting a more biologically reasonable temporal window for learning the response properties of inferior colliculus neurons in auditory midbrain; and second, by introducing hierarchical structure for learning the results of field recording in the auditory cortex. By using control experiments, we have found that max pooling in the hierarchical structure also plays an important role in producing the results.

Because the same principles, sparse coding and max pooling, are employed in different layers of the model, the results suggest that these principles might underlie computation at multiple stages of the auditory system. This is in agreement with the assumption that the auditory system evolved to optimize the representation of natural sounds [42], of which human speech is an example. Natural sounds contain many forms of higher-order and nonlinear statistical regularities [22, 42, 43], and sparse coding and max pooling are capable of extracting such regularities from natural images [28, 44, 45]. In this work, we demonstrate that these approaches can also extract them from human speech.

### Unsupervised learning for phoneme representation

Psychological studies have reported that infants are capable of distinguishing phonemes in their native language, even though they are not explicitly trained to accomplish this task [46, 47]. Inspired by this finding, many computational models have been proposed to investigate how phoneme categories can be formed from continuous speech through unsupervised learning (e.g., [48–50]). However, these single-layer models cannot reveal how phoneme information is encoded gradually along the auditory pathway. Aided by some side information, such as lexical information and the identity of the speaker, deep neural networks can learn phonetic features from speech [51, 52]. A critical step in these systems is time alignment of frames, which complicates the learning process and makes it hard to interpret the learning process of infants. Recently, two hierarchical models, the deep belief network and auto-encoder network, were trained on unlabeled speech corpus, but failed to obtain increasing phoneme selectivity in ascending layers [37]. By contrast, after supervised training with phoneme labels, the multilayer perceptron exhibited increasing selectivity to phoneme classes in higher layers [37], and its top layer exhibited a feature organization pattern similar to that of human auditory cortex [24]. Nevertheless, this supervised learning model cannot explain how phoneme representation in the human brain is developed during infancy. In this study, we showed that phonetic feature representation can emerge in an unsupervised learning model trained on continuous speech data without relying on any side information. Our results oppose the view that phoneme learning occurs after [51, 52] or concurrent with lexical learning and that the two processes cannot be addressed in isolation [53–55].

### Model predictions

The model makes two predictions that could be explored in future experiments. The first is that the emergence of selectivity for phoneme features along the auditory pathway is not abrupt; instead, it should be a continuous process. In our model, the selectivity emerged in several layers other than the top layer, although it was weaker in lower layers such as S5. Therefore, one may find neurons selective for phoneme features in subcortical areas, but their selectivity should not be as strong as in the auditory cortex. The second prediction is that there exist neurons in the auditory cortex that encode the formant dynamics of phonemes. As shown in Fig 7, some layer C6 units in the model were sensitive to variation in the first formant of the phonemes, the second formant, or both. This is parallel to the findings in the higher-level cortical areas in the visual pathway, in which neurons encode abstract features such as contours [56, 57]. The fact that such units were scarce in layers S5 to C5 suggests that neurons whose responses are correlated to formant dynamics are not common in subcortical areas.

### Limitations

This study has some limitations. First, because SHMAX is not a biologically detailed model, it is unclear how it could be implemented in a biological system. Some neural circuits have been proposed to individually realize the two essential components of the model, i.e., sparse coding [58] and max pooling [59, 60], but an approach for integrating them as a whole is still lacking. Second, this model differs from biological systems in many aspects. For example, real neurons can be excitatory or inhibitory, and they obey Dale’s law [61], but these features are not considered in SHMAX. In addition, the auditory system contains abundant feedback and recurrent connections, whereas SHMAX is a feedforward model. Due to these differences, the results in this paper should be interpreted cautiously. Although such discrepancies may not affect the abstract computational principles of the auditory system revealed by the model, a more biologically plausible model would make the results more convincing.

## Materials and methods

The model and analyses were implemented in MATLAB. The source code is available at https://github.com/QingtianZhang/SHMAX_AuditoryPathway.

### Stimuli

Speech stimuli from the Texas Instruments/Massachusetts Institute of Technology (TIMIT) database [62], which includes 6,300 sentences spoken by 630 speakers (10 sentences per speaker) from eight major dialect regions of the United States, were used. The sample rate is 16 kHz. The sentences were first converted into cochleogram [9, 63], which is similar to a spectrogram but better reflects the effects of the cochlea. A total of 194 frequency filters were generated from a cochlear model [9] whose center frequencies were between 73 and 7,630 Hz. The sample step was 1 ms. The cochleogram was used as the input for the SHMAX model.

### SHMAX

SHMAX [28] is an unsupervised deep learning model that integrates sparse coding into a well-known cortex-inspired visual recognition model, HMAX [64]. The structure used in this study (Fig 1) consists of six S layers and six C layers, which respectively perform sparse coding and max pooling, in alternation. We implemented the feedforward calculation of the model in the same manner as in a convolutional neural network [65, 66]. The major difference is that the convolutional kernels were not learned by supervised learning but by unsupervised learning, specifically sparse coding (see below). Another difference is that we did not use nonlinear activation functions as in standard convolutional neural networks. The details are as follows.

Sparse coding is an unsupervised learning technique inspired by sparse firing of V1 simple cells [20, 38]. Given a set of input signals ***x***^*k*^ ∈ *R*^*n*^, where *k* indexes the input, the objective of sparse coding is to find a set of bases ***b***_*j*_ ∈ *R*^*n*^ such that $x^{k}=\Sigma_{j=1}^{m}r_{j}^{k}b_{j}+\sigma^{k}$, where $r_{j}^{k}$ is the weighting factor of ***b***_*j*_ and ***σ***^*k*^ ∈ *R*^*n*^ is noise. The factor $r_{j}^{k}$ is called the “response” of neuron *j* to the *k*-th input, whose receptive field is delineated by ***b***_*j*_. The critical requirement of this technique is that $r_{j}^{k}$ values are sparse (i.e., only a few of them are nonzero). A standard formulation of sparse coding is

$${\mathrm{minimize}}_{B,r^{k}}\;\sum_{k}{\left\|x^{k}-Br^{k}\right\|}_{2}^{2}+\lambda {\left\|r^{k}\right\|}_{1}$$ (1)

$$subject\;to\;{\left\|b_{j}\right\|}_{2}^{2}\leq 1,\;j=1,2,\ldots ,m,$$

where ***B*** ∈ *R*^*n*×*m*^ is the collection of ***b***_*j*_, ***r***^*k*^ ∈ *R*^*m*^ is the collection of $r_{j}^{k}$, and *λ* is a constant controlling the tradeoff between the reconstruction error (the first term) and the sparseness (the second term). ‖⋅‖_2_ and ‖⋅‖_1_ stand for the L2-norm and L1-norm of vectors, respectively. All results reported in this paper, except in Fig 8, were obtained with *λ* = 1. Without loss of generality, it was assumed that the STRFs of all units in the model were square. The bases of sparse coding in each S layer were learned from a number of 10 ×10 × *u* patches extracted randomly from the input of that layer (therefore, the size of each basis was also 10 ×10 × *u*), where *u* denotes the number of the input channels. In layer S1, *u* was equal to 1, and in other S layers it was equal to the number of bases in the preceding C layer. The online dictionary learning algorithm was used to learn ***B*** [67].

The response of each S layer could be obtained by solving Eq (1) with learned ***B*** by inputting a sliding 10 ×10 × *u* patch in the previous layer as input [28]. This will result in a total of *m* feature maps consisting of responses of *m* bases ***b***_*j*_ at every location in the previous layer. In this study, we used another approach: each basis ***b***_*j*_ was convolved with the input to obtain the corresponding feature map. In doing so, ***b***_*j*_ was reshaped to 10 ×10 × *u* and convolved with the previous layer whose size was *h* × *t* × *u*, where *h* and *t* denote the height and width of the feature maps, then the *j*-th feature map (a 2D matrix) in the current layer was obtained as follows:

$$r_{S}(\hat{h},\hat{t},j)=\sum_{p=0}^{9}\sum_{q=0}^{9}\sum_{\hat{u}=0}^{u-1}x(s_{conv}\cdot \hat{h}+p,s_{conv}\cdot \hat{t}+q,\hat{u})\cdot b_{j}(p,q,\hat{u})$$ (2)

where *s*_*conv*_ is the convolution stride. This approach yielded similar results but was much faster. Note that the difference between the outputs of the two approaches is unimportant because the responses obtained by the convolution approach are also sparse (S7 Fig), and sparseness is the focus of this study. In addition, in the convolution approach it is natural to define ***b***_*j*_ as the receptive field of an artificial neuron because ***b***_*j*_ is used to explain the output, whereas in the optimization approach the correspondence between ***b***_*j*_ and receptive field is indirect because ***b***_*j*_ is used to explain the input.

The convolution stride *s*_*conv*_ was 2 in the first two S layers (equivalent to vanilla convolution followed by down-sampling with ratio 2) and 1 in the other four S layers (vanilla convolution). The value *u* in each S layer is indicated at the top of Fig 1C.

The C layers take the responses of S layers as input and perform the max pooling operation. We slide on the feature maps in an S layer and at each location take the maximum value in a region of size *s*_*pool*_ × *s*_*pool*_ centered at that location (for simplicity, square shapes of the regions are assumed). All maximum values taken in a feature map in the S layer then constitute a feature map in the subsequent C layer. Therefore, the number of feature maps in a C layer is equal to that in the preceding S layer. Clearly, the output (or response) of a C unit is the maximum response of some S units in a local region. The pooling results were input to the next S layer.

In this study, we used overlapping pooling (stride of 1), and the feature maps in a C layer had one fewer column and one fewer row than those in the preceding S layer. Specifically, the calculation is as follows:

$$r_{C}(\hat{h},\hat{t},j)=\max\left\{r_{C}(\hat{h}:\hat{h}+s_{pool}-1,\hat{t}:\hat{t}+s_{pool}-1,j\right\}$$ (3)

where *s*_*pool*_ = 2.

### Spectro-temporal receptive field (STRF)

The STRFs of the units in layer S1 were approximated by the corresponding bases. The STRFs of the units in higher layers were obtained by linearly combining the bases of the units in previous layers [28]. The logic is that a unit in the current layer is locally connected to the units in the previous layer, and the favorite input pattern (spectro-temporal receptive field, STRF) of this unit depends on the favorite input patterns of the units in the previous layer.

The bottom-up computation process is described as follows. First, it should be noted that the units in a given feature map share the same STRF. For a unit in the *j*-th feature map in layer S(*l*), where *l*>1, its STRF is defined as the weighted sum of the STRFs of the units in layer S(*l* − 1) with their centers aligned according to the locations of the weights in the basis ***b***_*j*_. If there is no down-sampling between layers S(*l*) and S(*l* − 1), this can be implemented by convolving ***b***_*j*_ with all of the *u*^*l*−1^ STRFs in layer S(*l* − 1). Note that the third dimension of ***b***_*j*_ is *u*^*l*−1^, the number of feature maps in layer S(*l* − 1), and the result is a 2D matrix, which is the STRF of the units in the *j*-th feature map in layer S(*l*). If there is a down-sampling operation with ratio *d* between layers S(*l*) and S(*l* − 1), one needs to first expand ***b***_*j*_ to 10*d* × 10*d* × *u*^*l*−1^, and then perform the convolution as described above. The nearest-neighbor interpolation was used for matrix expansion. See Fig 2A for an illustration of visualizing an S2 basis (STRF of an S2 unit). For a better illustration, in this example the first two dimensions of the S1 and S2 bases are assumed to be 3 and 2, respectively, instead of 10. Note that the weighted summation step in the figure is equivalent to 2D convolution (“full” mode) of the two input matrices. After obtaining STRFs of all S2 units, one can calculate STRFs of S3 units in the same way, and so forth.

One can also calculate STRFs of units in any S layer directly, without precomputing the STRFs of units in lower S layers (S1 Fig). This is equivalent to the bottom-up method (Fig 2A), except for some differences in the boundaries of STRFs. The latter method was used to visualize the STRFs of S units in this study.

Since a C unit takes the maximum response of four neighboring units in the preceding S layer (the max pooling ratio was 2) whose STRFs are the same, its STRF is very similar to the STRFs of the preceding S units except that it is a bit larger. Its size is jointly determined by the size of the STRFs of the four preceding S units and the shifts between them.

The STRF sizes of the units in each layer are shown in Table 2. Because all STRFs are assumed to be square, only the side lengths are shown in the table.

To test the validity of the visualization method described above, the STRFPak toolbox with the normalized reverse-correlation method [32, 33] was also used to calculate the STRFs of the units in layers S1, S2, and S3 (S3 Fig); this took much longer time than the linear combination method described above. The time lag used for calculating the stimulus auto-correlation was 200 ms, the tolerance value was 0.01, and the sparse parameter was 0. The overall mean firing rate was removed from the neuronal response, and the space-time non-separable algorithm was used.

To compare the model and experimental results [30, 34], singular value decomposition (SVD) was performed on the obtained STRFs, and the two unitary vectors corresponding to the first singular value were used to quantify the spectral and temporal response characteristics, namely the spectral and temporal profiles (Fig 3) [30]. The peaks of the spectral and temporal profiles determine the center frequency (Center F) and best temporal modulation frequency (Best T) respectively. The widths of the spectral and temporal profile that account for 90% of the total energy determine the bandwidth and the duration respectively.

### Phoneme selectivity index (PSI)

To calculate the responses of a computing unit to a particular phoneme in speech, the TIMIT phonetic transcriptions were used to align responses to the onsets of all instances of the phoneme. Phoneme length was not normalized. The maximum absolute value of the response of a unit along the phoneme duration was defined as that unit’s response amplitude. The PSI vectors [6] were employed to characterize the selectivity of the units to phonemes. The method is briefly outlined as follows, and more details can be found in Ref. [6]. For every unit, the distribution of its response amplitudes across all samples of each phoneme was estimated first. To calculate a unit’s PSI for a particular phoneme, the non-parametric Wilcox rank-sum test was used to determine whether the response amplitude distribution of the phoneme had a larger median than those of other phonemes (p<0.01). The number of phonemes whose median response amplitudes were statistically smaller than the median response amplitude of a particular phoneme was defined as the unit’s PSI for that phoneme. Because 33 phonemes were selected from the dataset, PSI ranges between 0 and 32, where 0 means no selectivity and 32 means extreme selectivity. The PSIs for all 33 phonemes form a 33-dimensional PSI vector for the unit.

### F-ratio

Active units whose responses to randomly selected time frames were statistically larger than their response to silence (p<0.001) were selected, and the F-ratio [24, 37] was used to quantify the overall phoneme selectivity of all active units in a layer (Table 1). The units were grouped based on the clustering of PSI vectors (see Fig 5C for an example). Suppose there are *m* active units in total, which form *n* groups in a certain layer. Let Ω_*j*_ denote the set of indices of active units in group *j*, and |Ω_*j*_| = *m*_*j*_. The F-ratio for that layer is defined as the ratio of between-group variability to within-group variability:

$$F=\frac{\sum_{j=1}^{n}m_{j}{\left|\left|{\overset{-}{p}}_{j}-\overset{-}{p}\right|\right|}_{2}^{2}/(n-1)}{\sum_{j=1}^{n}\sum_{i\in \Omega_{j}}{\left|\left|p_{i}-{\overset{-}{p}}_{j}\right|\right|}_{2}^{2}/(m-n)}$$ (4)

where ***p***_***i***_ denotes the PSI vector for unit *i*, $\overset{-}{p}$ denotes the average of PSI vectors over all units, and ${\overset{-}{p}}_{j}$ denotes the average of PSI vectors over group *j*. The larger the F-ratio, the better the clustering effect.

### Phoneme to feature transformation

Six distinctive features were used to describe the acoustic properties of each phoneme [68]. Each phoneme has only one of the six features. The PSI vectors of all phonemes that shared a particular feature were averaged to describe the feature selectivity of the units (Fig 5D).

### Phoneme acoustic parameter estimation

A series of static acoustic parameters were estimated for phonemes that play a perceptually important role in speech perception, including F0, F1, F2, VOT, and spectral peak. The values of the first three parameters were calculated as the median value of transcribed boundaries over the duration of the phoneme [6]. The VOT was extracted as the phoneme transcription boundary. The spectral peak was defined as the maximum energy along the frequency axis in the cochleogram. Acoustic parameters differ among individual instances of a phoneme; therefore, each acoustic parameter for each phoneme in the dataset is expressed as a distribution.

### Linear regression analysis

Similar to [6], a linear model *y* = ***wx*** + *b* was used to regress the static acoustic parameters *y* such as the fundamental frequency (F0), formant frequencies (F1, F2), voice-onset time (VOT), and spectral peak, based on the response amplitudes of the computing units ***x***, where ***w*** and *b* are parameters to be learned. The least-square error between the prediction *y* and the ground truth y* was minimized on a training set, and the trained model predicted the parameter values on a test set. The root-mean-squared error was calculated on the training set. A prediction for a test sample was regarded as “correct” if the prediction error was smaller than the root-mean-squared error. The percent of correct prediction on the test set was defined as the testing or decoding accuracy.

The acoustic parameters regressed were F0, F1, and F2 of vowels and VOT and spectral peak of consonants. Fig 6A and 6B presents the decoding results using the responses of all active units in layer C6. Fig 6C and 6D presents the decoding results using the responses of active units, in each of the six phoneme groups separately, in different layers. For each task, a 20-fold cross-validation scheme was adopted, and therefore 20 decoding accuracies were obtained. To conduct a significance test, a random decoder was constructed. Given a test sample, the decoder output a value between the minimum and maximum values of the ground truth y* on the training samples, with a uniform probability. This random prediction was evaluated as correct or not based on the same criterion used for linear prediction. The 20-fold cross-validation scheme resulted in 20 chance-level accuracies. Student’s t-test was performed to compare the two sets of accuracies.

### Temporal variation index of phoneme formants

The time course of the first formant frequency of a phoneme instance was called the F1 contour of that instance (Fig 7A). The averaged F1 contours over all instances of a phoneme was defined as the F1 contour of the phoneme. Since 33 phonemes were selected in the dataset, we obtained 33 F1 contours. Principal component analysis (PCA) was performed on these contours. The first principal component, which had the same length as the F1 contour, was calculated (Fig 7B). The projection of the F1 contour of each phoneme onto the first principal component (a scalar) was defined as the F1 temporal variation index (TVI) of that phoneme. A unit’s average response to all instances of a phoneme (a scalar) was defined as its response to that phoneme. The encoding of F1 dynamics in a unit was measured as the correlation between the unit’s responses to all of the 33 phonemes and the F1 TVIs of those phonemes. The same procedure was applied to measure the encoding of F2 dynamics in a unit.

### Calculation of lifetime sparseness

The definition of lifetime sparseness of a unit is as follows [39]:

$$S=1-\frac{{\left(E[r]\right)}^{2}}{E[r^{2}]}$$ (5)

where *r* denotes the response of the unit and the expectation is taken across all test data.

## Supporting information

S1 Fig Calculation of the STRFs of S units using the linear combination method. A top–down method was used to calculate the STRF of any basis in layer S(*l*). Suppose that the third dimension of the basis is *u*^*l*−1^. The basis was first expanded and padded, which is the pseudo inverse operation of down-sampling and pooling. Then, the basis was convolved with all bases in layer S(*l* − 1) to obtain a 3D matrix whose third dimension is *u*^*l*−2^. By repeating this process until *l* = 1, we get the STRF of the specific basis in layer S(*l*). In the figure, *p* denotes the height and weight of the patch, *s*_*conv*_ is the convolution stride, and *l* ≥ 2 denotes the layer index. Note that, when *l* = 2, *u*^*l*−2^ = 1, and the process is the same as in Fig 2A. When *l* > 2, *u*^*l*−2^ > 1, and after 2D convolution and summation, one obtains a 3D matrix instead of a 2D matrix.
(TIF)

S2 Fig STRFs of all units in layers S1 (a), S2 (b), and S3 (c) of the SHMAX model.
(TIF)

S3 Fig **STRFs of all units in layers S2 (left) and S3 (right) using the reverse-correlation method.** The results are similar to those obtained using the linear combination method (S2 Fig).
(TIF)

S4 Fig PSI vectors of active units in Layer S3 (a) and Layer S4 (b).
(TIF)

S5 Fig PSI vectors of active units in Layer S5 (a), Layer C5 (b) and Layer S6 (c).
(TIF)

S6 Fig Influence of the number of layers and STRF size on PSI vectors. (a) PSI vectors of the new layer S5 with larger kernel size 20×20, obtained by fixing layers S1 to C4 of the original network. (b) PSI vectors of the new layer S6 with smaller kernel size 5×5, obtained by fixing layers S1 to C5 of the original network. (c) PSI vectors of the new layer S7 with kernel size 5×5, obtained by fixing layers S1 to C5 of the original network and use the layer S6 with smaller kernel size 5×5.
(TIF)

S7 FigResponse statistics obtained by convolving different kinds of bases on the feature maps in layer C4 across ten randomly selected sentences.(a) Response distribution of a layer S5 basis learned by sparse coding. The Kolmogorov–Smirnov (KS) test showed that the response followed a Laplacian distribution (p<0.05), which is sparse. The dashed lines show the fitted results with a zero mean Laplacian distribution whose probability density function is $f(x)=\frac{1}{2\sigma }\exp\left(-\frac{\left|x\right|}{\sigma }\right)$, where σ is the fitting parameter. Note that the vertical axis is in log-scale. (b) Response distribution of all layer S5 bases learned by sparse coding. The distribution is also very sparse. (c) Distribution of the element value in all layer S5 bases. (d) Response distribution of a basis whose elements were randomly sampled from the distribution in (c). The KS test showed that the response followed a Gaussian distribution (p<0.05), which is dense. The dashed lines show the fitted results with a zero mean Gaussian distribution whose probability density function is $f(x)=\frac{1}{\sqrt{2\pi }\sigma }\exp\left(-\frac{x^{2}}{2\sigma^{2}}\right)$, where σ is the fitting parameter.(TIF)
