# Supplementary figures and images for: A hierarchical sparse coding model predicts acoustic feature encoding in both auditory midbrain and cortex
Source: PLoS Comput Biol. 2019 Feb 11;15(2):e1006766. doi: 10.1371/journal.pcbi.1006766 (PMC6386396; doi:10.1371/journal.pcbi.1006766)

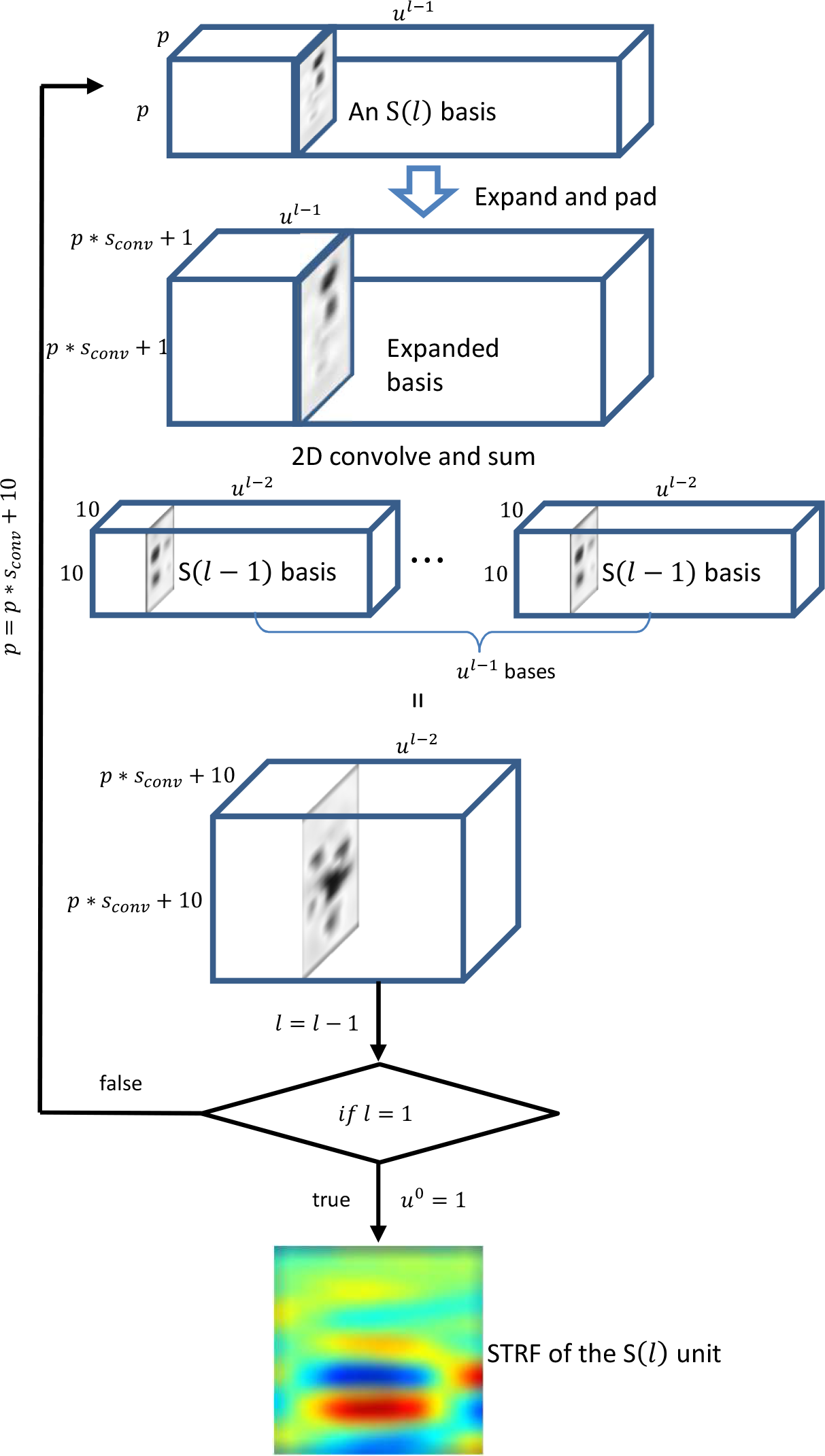

Supplement: S1 Fig — A top–down method was used to calculate the STRF of any basis in layer S(l). Suppose that the third dimension of the basis is ul−1. The basis was first expanded and padded, which is the pseudo inverse operation of down-sampling and pooling. Then, the basis was convolved with all bases in layer S(l − 1) to obtain a 3D matrix whose third dimension is ul−2. By repeating this process until l = 1, we get the STRF of the specific basis in layer S(l). In the figure, p denotes the height and weight of the patch, sconv is the convolution stride, and l ≥ 2 denotes the layer index. Note that, when l = 2, ul−2 = 1, and the process is the same as in Fig 2A. When l > 2, ul−2 > 1, and after 2D convolution and summation, one obtains a 3D matrix instead of a 2D matrix. (TIF) [file pcbi.1006766.s001.tif]

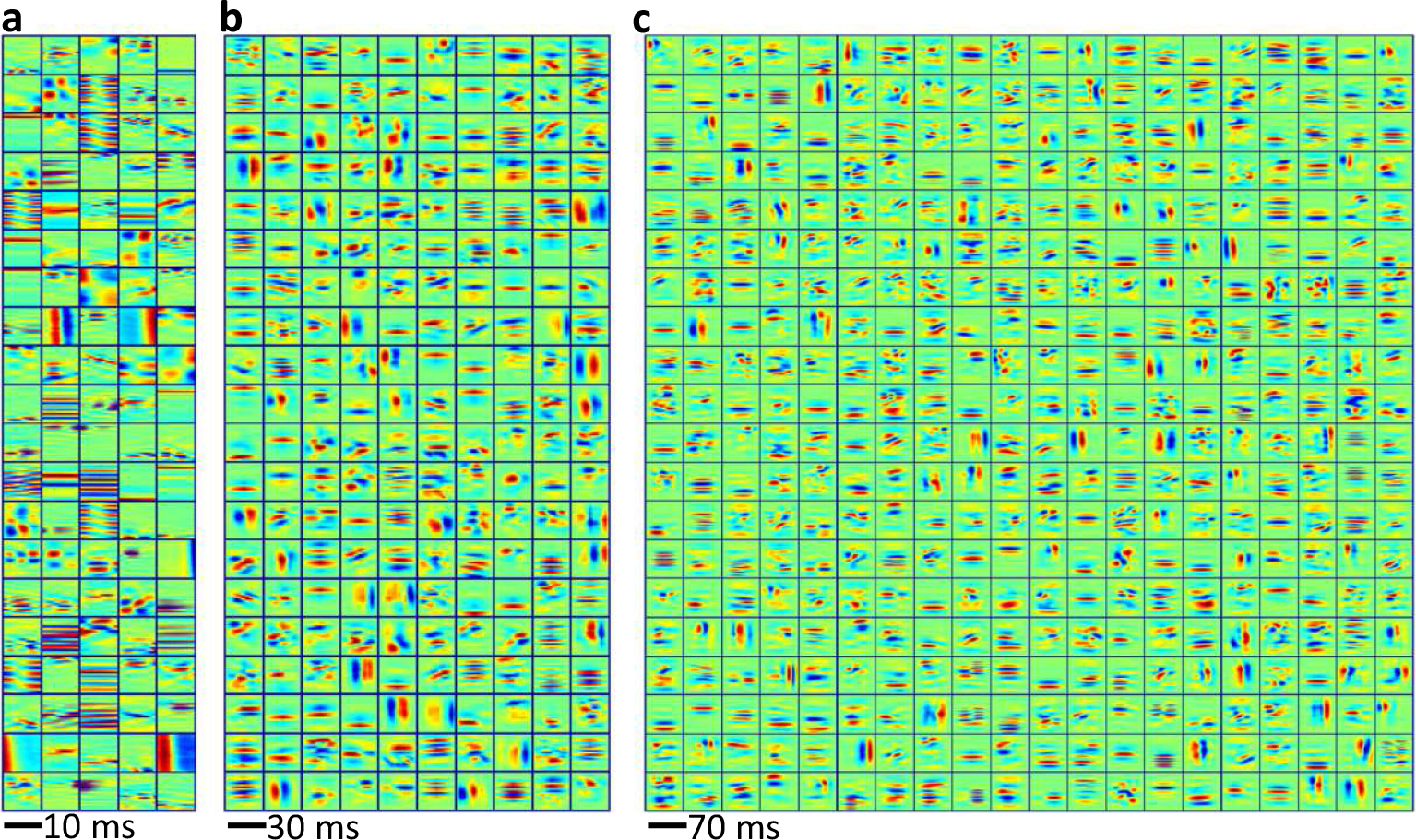

Supplement: S2 Fig — STRFs of all units in layers S1 (a), S2 (b), and S3 (c) of the SHMAX model. (TIF) [file pcbi.1006766.s002.tif]

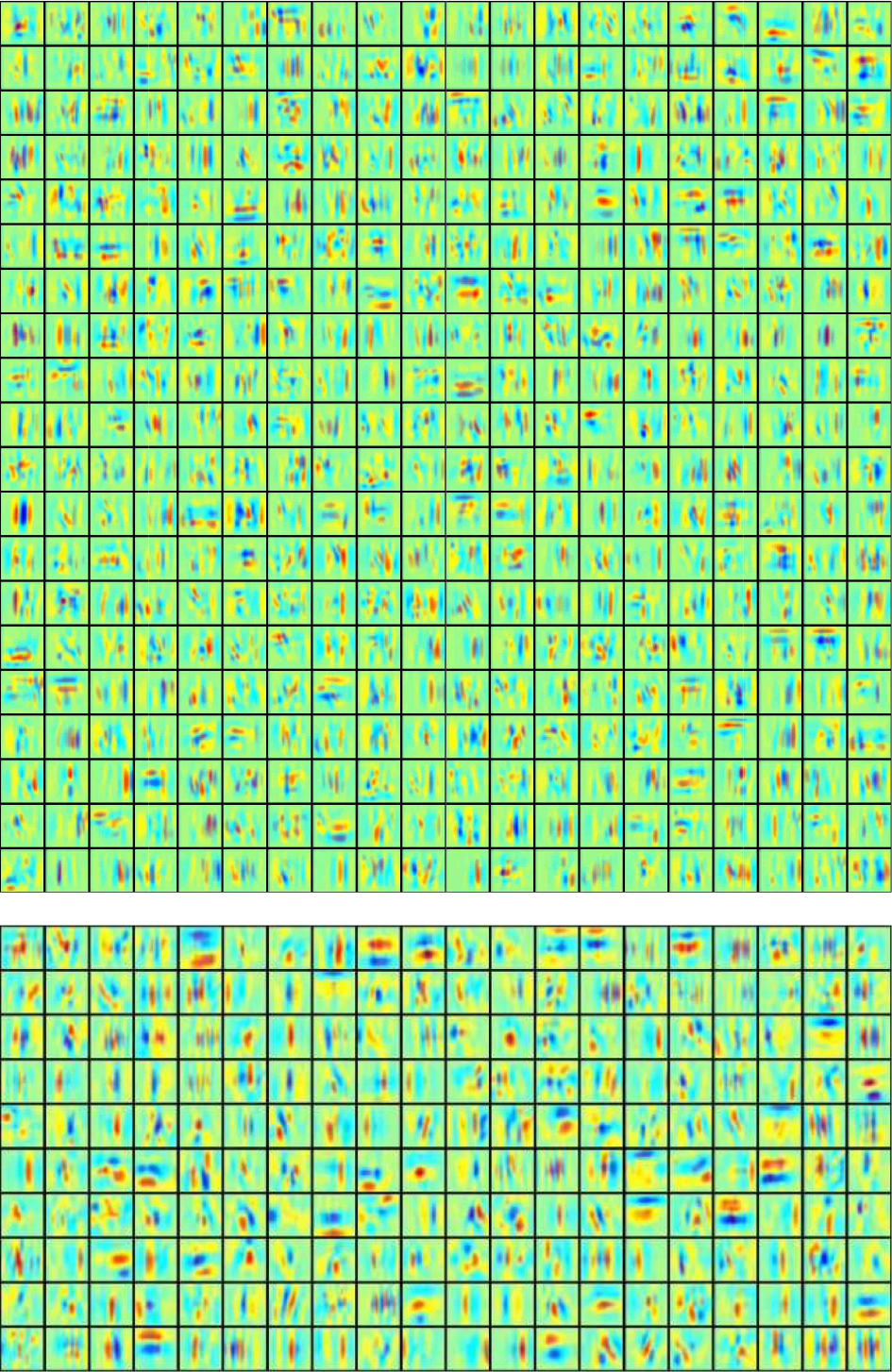

Supplement: S3 Fig — STRFs of all units in layers S2 (left) and S3 (right) using the reverse-correlation method. The results are similar to those obtained using the linear combination method (S2 Fig). (TIF) [file pcbi.1006766.s003.tif]

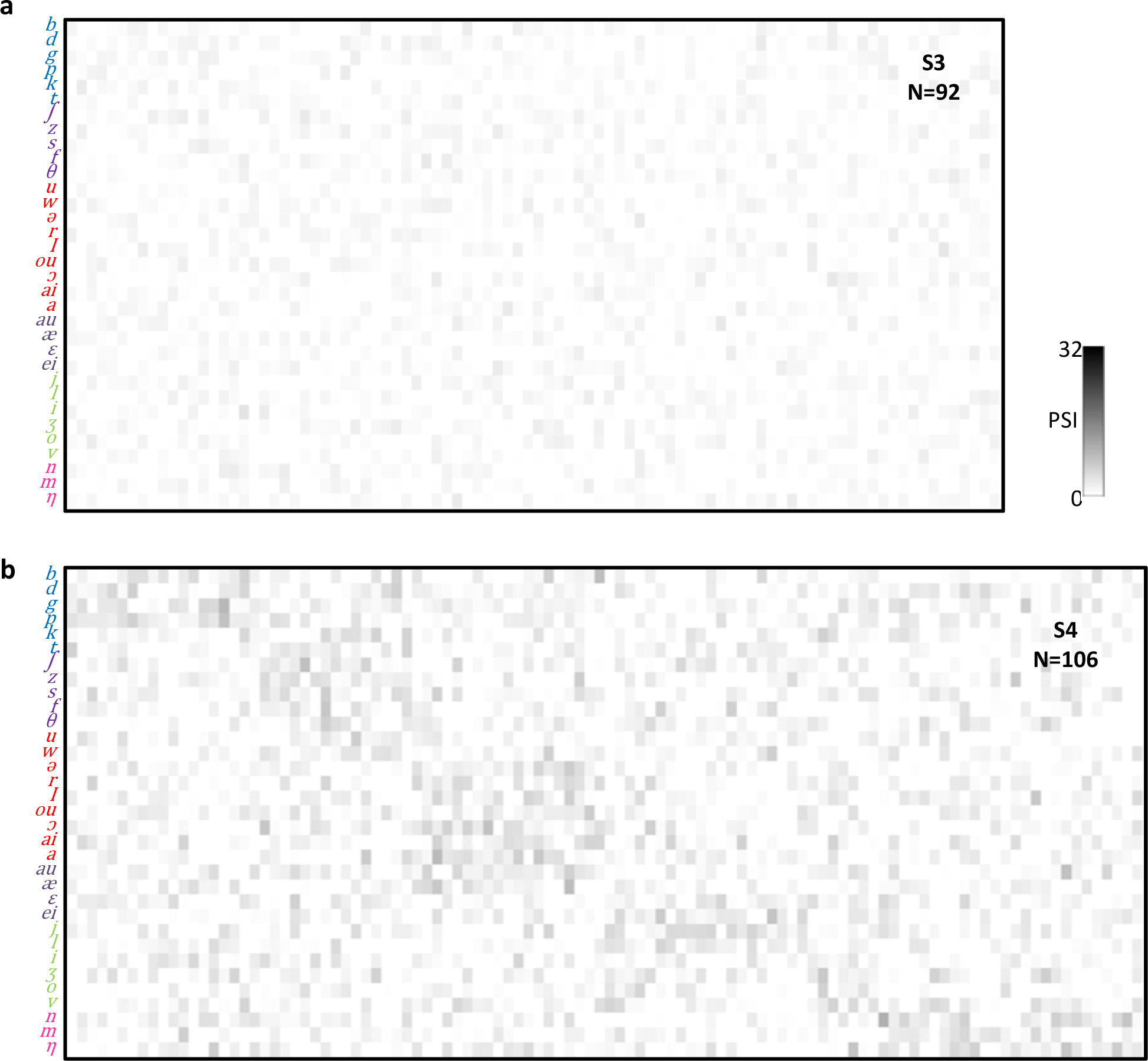

Supplement: S4 Fig — PSI vectors of active units in Layer S3 (a) and Layer S4 (b). (TIF) [file pcbi.1006766.s004.tif]

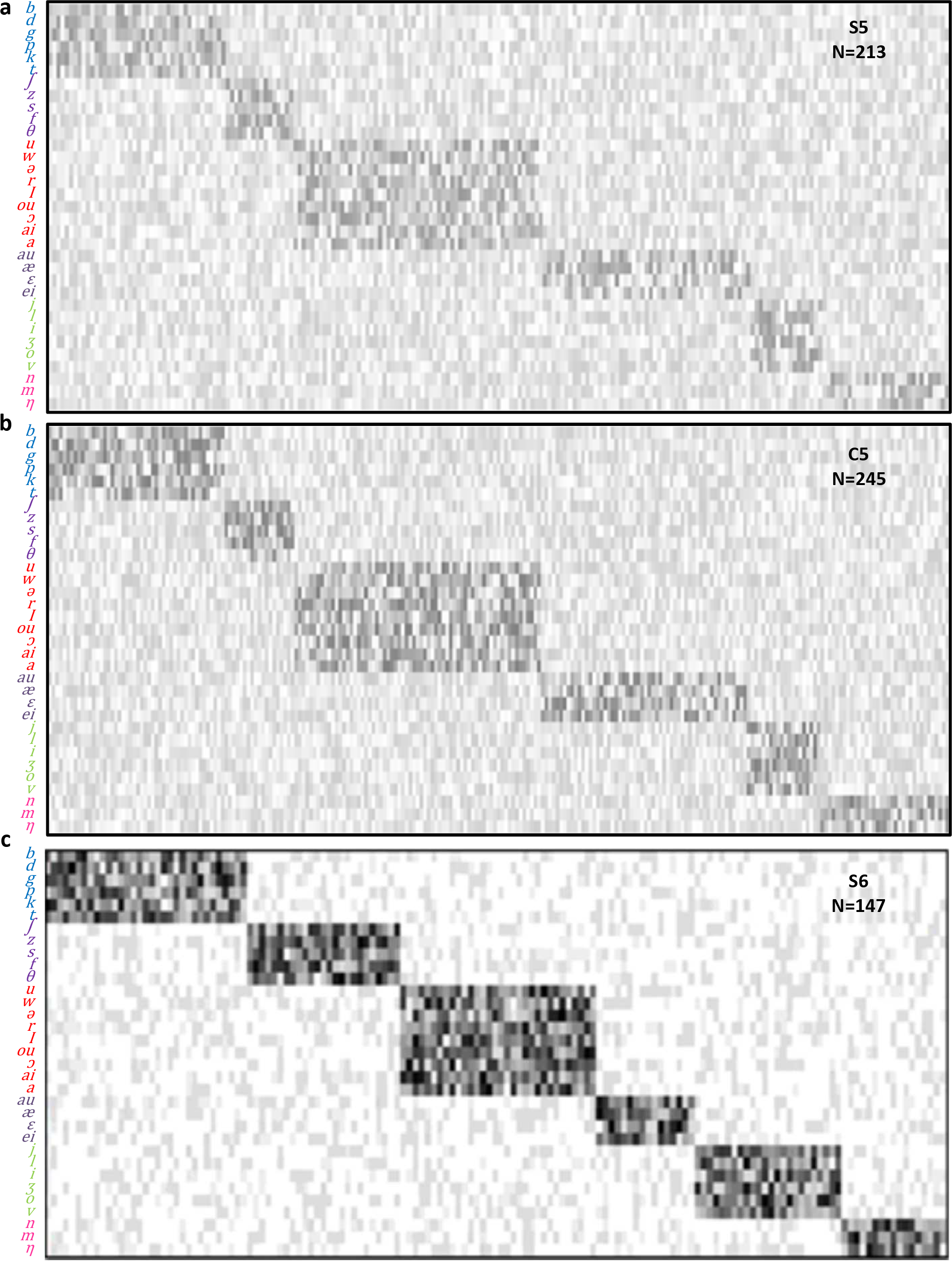

Supplement: S5 Fig — PSI vectors of active units in Layer S5 (a), Layer C5 (b) and Layer S6 (c). (TIF) [file pcbi.1006766.s005.tif]

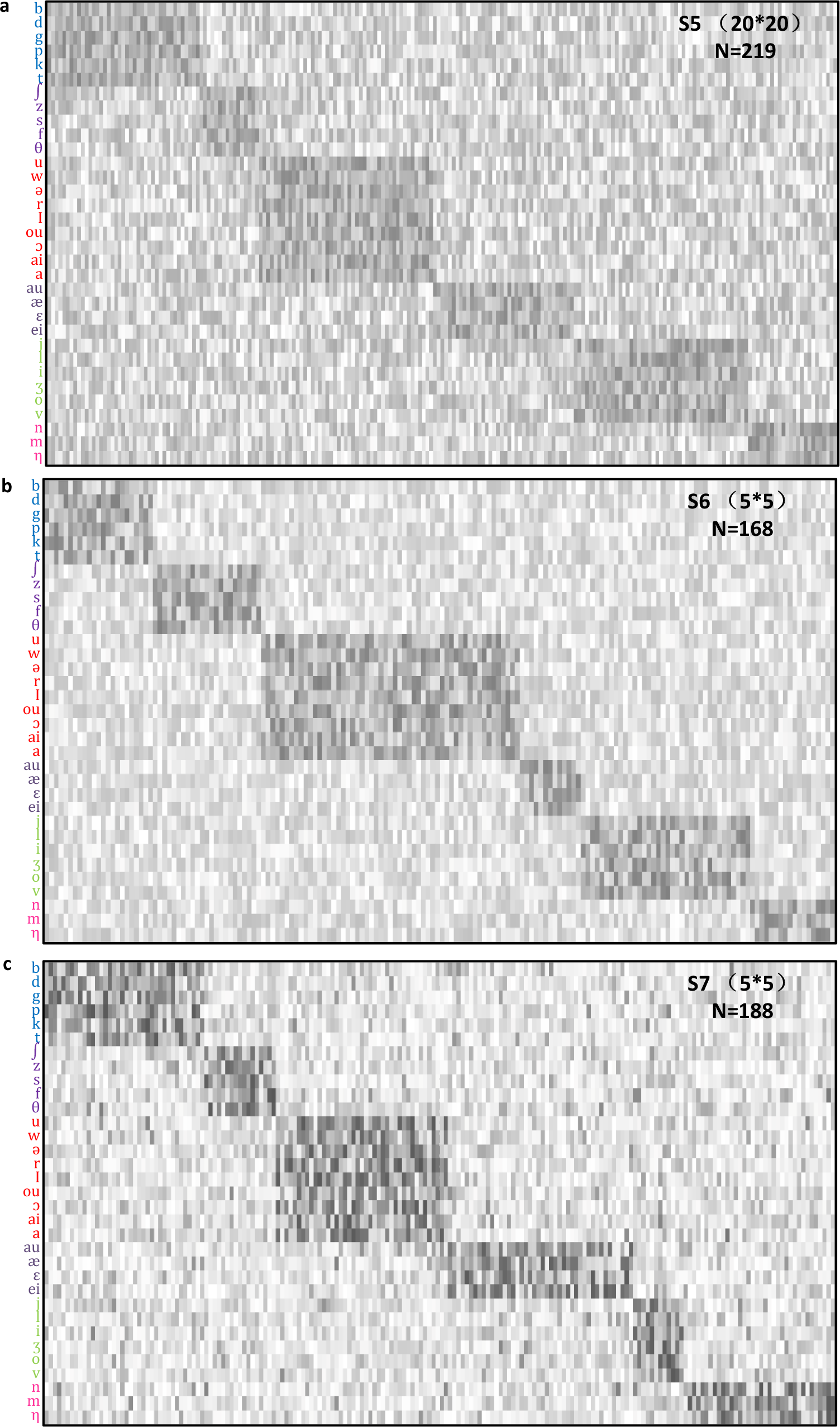

Supplement: S6 Fig — (a) PSI vectors of the new layer S5 with larger kernel size 20×20, obtained by fixing layers S1 to C4 of the original network. (b) PSI vectors of the new layer S6 with smaller kernel size 5×5, obtained by fixing layers S1 to C5 of the original network. (c) PSI vectors of the new layer S7 with kernel size 5×5, obtained by fixing layers S1 to C5 of the original network and use the layer S6 with smaller kernel size 5×5. (TIF) [file pcbi.1006766.s006.tif]

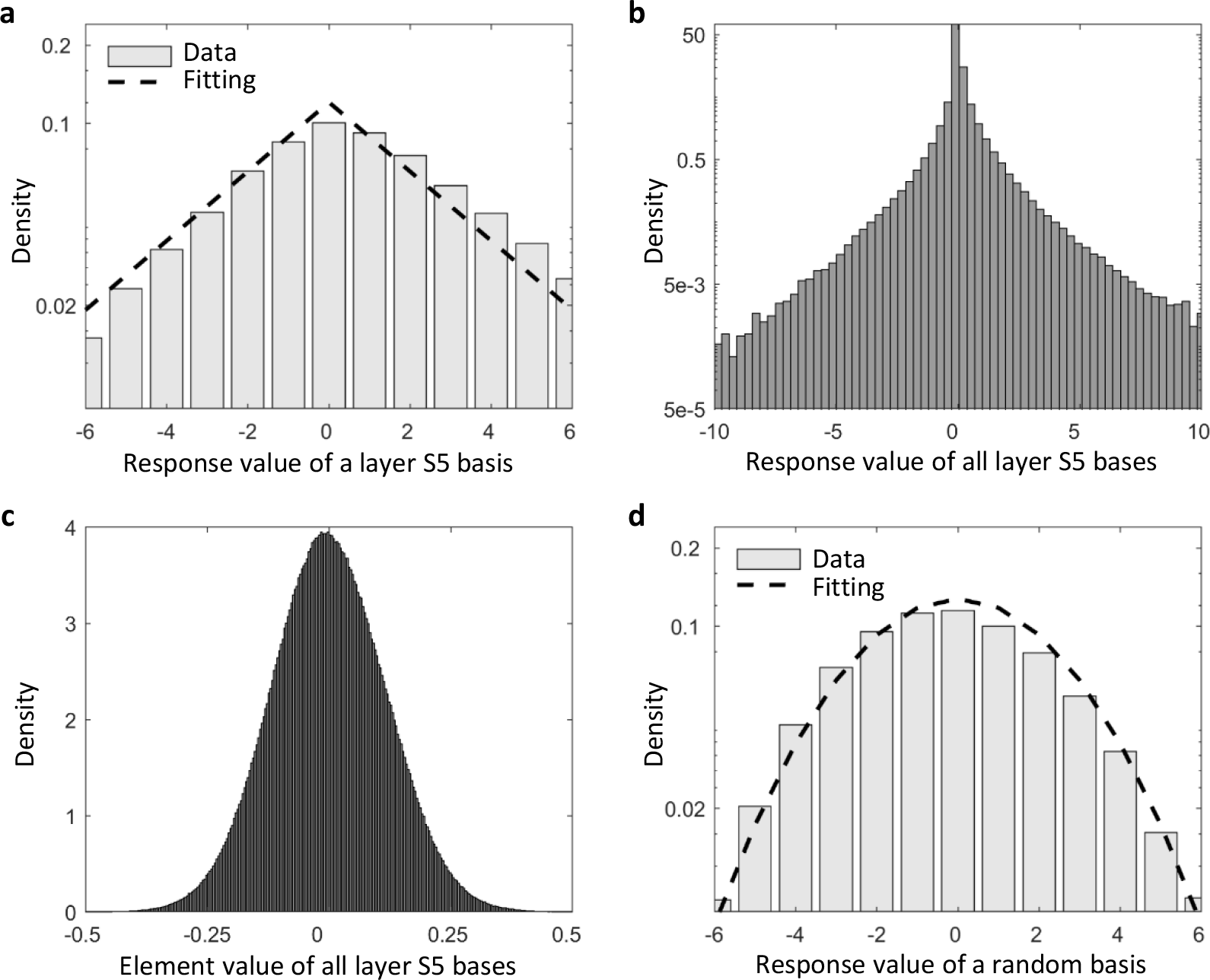

Supplement: S7 Fig — (a) Response distribution of a layer S5 basis learned by sparse coding. The Kolmogorov–Smirnov (KS) test showed that the response followed a Laplacian distribution (p<0.05), which is sparse. The dashed lines show the fitted results with a zero mean Laplacian distribution whose probability density function is f(x)=12σexp(−|x|σ), where σ is the fitting parameter. Note that the vertical axis is in log-scale. (b) Response distribution of all layer S5 bases learned by sparse coding. The distribution is also very sparse. (c) Distribution of the element value in all layer S5 bases. (d) Response distribution of a basis whose elements were randomly sampled from the distribution in (c). The KS test showed that the response followed a Gaussian distribution (p<0.05), which is dense. The dashed lines show the fitted results with a zero mean Gaussian distribution whose probability density function is f(x)=12πσexp(−x22σ2), where σ is the fitting parameter. (TIF) [file pcbi.1006766.s007.tif]
